# Akt3-Mediated Protection Against Inflammatory Demyelinating Disease

**DOI:** 10.3389/fimmu.2019.01738

**Published:** 2019-07-25

**Authors:** Juwen C. DuBois, Alex K. Ray, Ross C. Gruber, Yongwei Zhang, Ranee Aflakpui, Fernando Macian-Juan, Bridget Shafit-Zagardo

**Affiliations:** ^1^Department of Pathology, Montefiore Medical Center, Albert Einstein College of Medicine, Bronx, NY, United States; ^2^Department of Microbiology and Immunology, Montefiore Medical Center, Albert Einstein College of Medicine, Bronx, NY, United States; ^3^Multiple Sclerosis and Neuroinflammation Research, Sanofi, Framingham, MA, United States; ^4^Department of Cell Biology, Montefiore Medical Center, Albert Einstein College of Medicine, Bronx, NY, United States

**Keywords:** Akt3, neuroprotection, neuroinflammation, demyelination, EAE, regulatory T-cells (Tregs)

## Abstract

Akt is a serine/threonine protein kinase that plays a major role in regulating multiple cellular processes. While the isoforms Akt1 and Akt2 are involved in apoptosis and insulin signaling, respectively, the role for Akt3 remains uncertain. Akt3 is predominantly expressed in the brain, and total deletion of Akt3 in mice results in a reduction in brain size and neurodegeneration following injury. Previously, we found that Akt3^−/−^ mice have a significantly worse clinical course during myelin-oligodendrocyte glycoprotein (MOG)-induced experimental autoimmune encephalomyelitis (EAE), an animal model in which autoreactive immune cells enter the CNS, resulting in inflammation, demyelination, and axonal injury. Spinal cords of Akt3^−/−^ mice are severely demyelinated and have increased inflammation compared to WT, suggesting a neuroprotective role for Akt3 during EAE. To specifically address the role of Akt3 in neuroinflammation and maintaining neuronal integrity, we used several mouse strains with different manipulations to Akt3. During EAE, Akt3^*Nmf350*^ mice (with enhanced Akt3 kinase activity) had lower clinical scores, a lag in disease onset, a delay in the influx of inflammatory cells into the CNS, and less axonal damage compared to WT mice. A significant increased efficiency of differentiation toward FOXP3 expressing iTregs was also observed in Akt3^*Nmf350*^ mice relative to WT. Mice with a conditional deletion of Akt3 in CD4^+^ T-cells had an earlier onset of EAE symptoms, increased inflammation in the spinal cord and brain, and had fewer FOXP3^+^ cells and *FOXP3* mRNA expression. No difference in EAE outcome was observed when Akt3 expression was deleted in neurons (Syn1-CKO). These results indicate that Akt3 signaling in T-cells and not neurons is necessary for maintaining CNS integrity during an inflammatory demyelinating disease.

## Introduction

The Akt family of serine/threonine protein kinases, also known as protein kinase b (PKB), consists of three isoforms Akt1, Akt2, and Akt3. Although the Akt isoforms share a high degree of structural homology and amino acid identity, they are encoded by three separate genes and are not able to functionally compensate for one another. In addition, the expression of each isoform varies by tissue type. Akt1 is most abundantly expressed in the majority of tissues, Akt2 expression is highest in the skin and insulin-responsive tissues such as brown fat, skeletal muscle, and liver, whereas Akt3 is predominant in the brain (1–4).

While Akt1 and Akt2 are involved in a variety of cellular processes including cell survival, cell proliferation, apoptosis, differentiation, and insulin signaling, the specific role of Akt3 remains largely unexplored (5). Akt3 is the major Akt isoform in neurons and represents 50% of the total Akt in the brain, and 30% of that in the spinal cord (2). Akt3 is significantly decreased in the normal appearing white matter (NAWM) of individuals with multiple sclerosis (MS) relative to NAWM from healthy controls (6).

Mice lacking Akt1 (Akt1^−/−^) have an overall decrease in body size and growth, and constitutively active Akt1 expression enhances remyelination in mice (1, 3, 7). Akt2^−/−^ mice display a diabetes-like phenotype with insulin intolerance and a mild growth deficiency. However, Akt3^−/−^ mice have a ~25% reduction in brain size, with smaller and fewer cells, and enhanced Akt3 kinase activity in Akt3^*Nmf350*^ mice results in a ~20–25% increase in brain size and ectopic hippocampal neurons (1–4, 8–12).

Currently, little is known about the genes that are regulated by Akt3 and there are few identified Akt3 substrates. Lack of Akt3 expression in mice results in a more severe clinical course during myelin-oligodendrocyte glycoprotein (MOG)-induced experimental autoimmune encephalomyelitis (EAE). We observed an earlier disease onset, higher clinical scores, more axonal damage, and increased demyelination compared to WT mice during both acute and chronic disease phases. Akt3^−/−^ mice have more CD45^+^ and Iba1^+^ cells in the spinal cord, and upregulate the mRNA expression of proinflammatory cytokines *IL-2, IL-17*, and *IFN-*γ relative to WT mice, suggestive of a role of Akt3 in conferring protection to mice during EAE (13). Akt3^−/−^ mice also have significantly reduced levels of MAP-2 protein and more SMI32^+^ swellings indicative of neuronal damage during acute EAE. Additionally, WT mice receiving donor bone marrow cells from Akt3^−/−^ mice had significantly higher clinical scores during EAE than control mice receiving bone marrow cells from WT mice; suggesting a functional role for Akt3 in peripheral immune cells.

Akt signaling is critical for the development and function of T-cells, and Akt is known to regulate several downstream transcription factors including NF-κB and FOXO3 that are involved in T-cell activation (14, 15). Akt contributes to T-cell function through its involvement in T-cell development, differentiation, and activation; however, the T-cell specific roles of individual Akt isoforms have largely been overlooked (16).

Regulatory T-cells (Tregs) specialize in suppressing and controlling inflammation and preventing unwanted cellular damage caused by overactive immune responses. Studies have found that mice with constitutively active Akt1 have a less severe EAE course, enhanced regulatory T-cell (Treg) differentiation, and an impaired Th17 response (17). While we have determined that Akt3 is expressed in CD4^+^ T-cells, its expression affects the ability of Th1 and Th17 cells to be suppressed by Tregs (13). In addition, Akt3 has been shown to affect the double-negative to double-positive transition during thymocyte T-cell development (18). The differentiation of CD4^+^ T-cell lineages is mediated by cytokines that initiate different transcriptional programs that may be regulated by AKT-dependent lineage-specific transcription factors.

In addition to its role in EAE and T-cells, Akt3 has been implicated in providing neuronal protection during neuronal injury, and is involved in post-natal brain development (4). Akt3 is significantly upregulated during spinal cord injury in rats, is neuroprotective in a mouse model of Familial Amyotrophic Lateral Sclerosis (ALS) and EAE, and has been identified as the most prominent Akt isoform involved in the protection against neurodegeneration (13, 19–21). Thus, we initiated studies to determine the cell-specific role of Akt3 in the CNS that regulates susceptibility to MOG-induced EAE. Our goal was to test the hypothesis that lack of Akt3 in T-cells and neurons results in a more severe disease course, since Akt3 may be necessary for proper CNS and T-cell function.

## Materials and Methods

### Mouse Strains

All procedures were approved by the Institute of Animal Care and Use Committee at Albert Einstein College of Medicine in compliance with the National Institutes of Health's Guide for Care and Use of Laboratory Animals. All mice used were on a C57Bl/6J background. Wild-type (WT), CD4-Cre, and Synapsin1-Cre (Syn1-Cre) mice were purchased from Jackson Laboratories (Bar Harbor, ME) and bred in-house at Albert Einstein College of Medicine. Akt3^−/−^ mice were obtained from Dr. Morris Birnbaum (University of Pennsylvania School of Medicine, Philadelphia, PA). Akt3^*Nmf350*^ mice were obtained from Dr. Wayne Frankel at Jackson Laboratories, Bar Harbor, ME, and extensively backcrossed onto a C57Bl/6J background. Conventional PCR and Sanger sequencing were performed to determine zygosity of the Akt3^*Nmf350*^ mutation found in Akt3 exon 8.

### Generation of Akt3 Conditional Knockout Mice

Akt3 conditional knockout (CKO) mice were generated using CRISPR/Cas9 technology by inserting loxP sequences flanking Akt3 exon 3. CKO mice were generated by Dr. Yongwei Zhang in collaboration with Dr. Winfried Edelmann at the Gene Targeting and Transgenic Facility, Albert Einstein College of Medicine. Guide RNAs (gRNA) were designed to target intron 2 (GAGCCCATCTTCAGTCTGAC) and intron 3 (CTTGCATGTTTAACTAGGGCTGG) of murine Akt3 using the CRISPR Online Design tool (Zhang Lab, MIT; http://crispr.mit.edu). gRNAs were generated by *in vitro* transcription (22). Cas9 mRNA was purchased from Systems Bioscience (Palo Alto, CA). Akt3 Homologous Recombination Donor (HRD) plasmid containing the 2 kb homologous arms at each side and the floxed exon 3 was generated by SLiCE (23).

Super ovulated female C57Bl/6J mice (3–4 weeks old) were mated to C57Bl/6J males, and fertilized embryos were collected from oviducts. *In vitro* transcribed guide RNAs (gRNAs) targeting to intron 2 and intron 3 of Akt3, Cas9 mRNA, and Akt3 conditional knockout HRD were mixed and microinjected into the cytoplasm of fertilized eggs. The injected zygotes were transferred into pseudopregnant CD1 females. A male Akt3^fl/+^ mouse was obtained from the resulting pregnancy. The founder mouse was mated with 5 WT females from separate mating lines; the resulting offspring were mated to generate Akt3^fl/fl^ mice. LoxP zygosity was determined using conventional PCR and gel electrophoresis using primers spanning the loxP insertion sites (primers listed in Table 1). WT alleles migrated at 374 bp, homozygous alleles migrated at 424 bp, and hemizygous bands migrated at both 374 and 424 bp. Genotyping was performed by GeneTyper (New York, NY).

**Table 1:** Genotyping primer sequences.

**Strain**	**Primers and probes**
Akt3*^Nmf*350*^*	Forward: AGTGCTCATCACAACTACCAAA
	Reverse: GCCATCTTTGTCTACTCAACAGG
CD4-CKO	Forward: GCCTTTGTAAGTCACAGAAAGTAGCT
	Reverse: TTGCCCAACAGATCCACTAAGTC
	Probe: CTGTTCTAGGACCAATCTA
Syn1-CKO	Forward: TTAATCCATATTGGCAGAACGAAAACG
	Reverse: CAGGCTAAGTGCCTTCTCTACA
	Probe: CCTGCGGTGCTAACC
Akt3^fl/fl^	Forward: TCAGAACCCTTCCCATTTGGT
	Reverse: CCAGAGGAAAGGTAAGAGACTTG

Conditional knockout of Akt3 in CD4^+^ T cells was done by mating Akt3^fl/fl^ mice with CD4-Cre mice until mice had a CD4-Cre^+^ Akt3^fl/fl^ genotype (CD4-CKO mice). Conditional knockout of Akt3 in neurons was done by mating Akt3^fl/fl^ mice with Syn1-Cre mice until mice had a Syn1-Cre^+^ Akt3^fl/fl^ genotype (Syn1-CKO mice). Mouse matings were established to only produce mice hemizygous for Cre. Genotyping for CD4-Cre and Syn1-Cre was performed by Transnetyx, Inc. (Cordova, TN) by real-time PCR using the primers and probes listed in Table 1. A positive signal indicated the presence of the Cre allele. Experimental controls (WT) included C57Bl/6J mice, CD4Cre mice, Akt3^fl/fl^ mice, and SynCre mice. We observed no difference in EAE outcome between these groups. The appropriate WT controls used for each study is described in the figure legend. For CKO studies the Cre- and Akt3^fl/fl^ control mice yielded similar results.

### Induction of EAE

MOG-induced EAE was conducted in sex-matched adult mice between the ages of 8–12 weeks. Mice were immunized with MOG_35−55_ peptide (MEVGWYRSPFSRVVHLYRNGK, synthesized by CelTek Bioscience, Nashville, TN) on day 0. MOG_35−55_ (3 mg/mL) was emulsified in an equal volume of complete Freund's adjuvant (CFA) and 100 μL of emulsion was injected subcutaneously on each hind flank (200 μL total/mouse). Pertussis toxin (Ptx, 2.5 μg/mL; List Biological Laboratories, Campbell, CA) was administered intraperitoneally on day 0 and day 2 (200 μL/injection). Mice were monitored daily for clinical symptoms and were scored as follows: 0 = no clinical symptoms, 1 = flaccid tail, 2 = flaccid tail and hind limb weakness, 3 = hind limb paralysis, 4 = hind limb paralysis and forelimb weakness, 5 = moribund. Mice that did not present with clinical symptoms were excluded from analysis. Acute EAE is defined as mice having clinical symptoms of disease (CI ≥ 1) for 4–5 consecutive days, and chronic EAE is defines as mice having clinical symptoms of disease (CI ≥ 1) for >10 consecutive days.

### Real-Time Quantitative RT-PCR

Total RNA was extracted from brain and spinal tissue using TRIzol reagent (ThermoFisher Scientific, Waltham, MA). For brain sections, RNA was extracted from a 2 mm section of corpora callosa. RNA was quantified using a NanoDrop ND-2000 spectrophotometer (ThermoFisher) at an absorbance ratio of 260 and 280 nm, and 1 μg RNA was used for cDNA synthesis. Reverse transcription was carried out using iScript cDNA Synthesis Kit (Bio-Rad, Hercules, CA). For qRT-PCR reactions, templates were diluted 1:5 in a PCR mix containing each gene-specific primer pair and iTaq Universal SYBR Green Supermix (Bio-Rad). Gene expression was measured on a StepOne Plus Real-Time PCR system (Applied Biosystems). All samples were run in duplicate/triplicate and normalized to the geometric mean of β-actin, HPRT, or GAPDH with a WT sample as reference. Melting curves were analyzed for each sample to confirm specificity of the amplicon. Fold change was determined using the 2^−ΔΔCt^ method (24). Primers for the genes analyzed are listed in Table 2.

**Table 2:** qRT-PCR primer list.

**Gene**	**Primers**
TNF-α	Forward: TGTAGCCCACGTCGTAGCAA
	Reverse: AGGTACAACCCATCGGCTGG
IFN- γ	Forward: AAAGAGATAATCTGGCTCTGC
	Reverse: GCTCTGAGCAATGAACGT
IL-1β	Forward: TGTGCAAGTGTCTGAAGCAGC
	Reverse: TGGAAGCAGCCCTTCATCTT
IL-2	Forward: CCCAAGCAGGCCACAGAATTGAAA
	Reverse: TGAGTCAAATCCAGAACATGCCGC
IL-4	Forward: GGTCTCAACCCCCAGCTAGT
	Reverse: GCCCGATGATCTCTCTCAAGTGAT
IL-6	Forward: ATTGGATGCTTACCAAACTGGAT
	Reverse: TGAAGGACTCTGGCTTTGTCT
IL-10	Forward: GCTCTTACTGACTGGCATGAG
	Reverse: CGCAGCTCTAGGAGCATGTG
IL-13	Forward: CCTGCCTCTTGCTTGCCTT
	Reverse: GGTCTTGTGTGATGTTGCTCA
IL-17	Forward: CAGCAGCGATCATCCCTCAAAG
	Reverse: CAGGACCAGGATCTCTTGCTG
TGF-β	Forward: ACCATGCCAACTTCTGTCTG
	Reverse: CGGGTTGTGTTGGTTGTAGA
T-bet	Forward: AGCAAGGACGGCGAATGTT
	Reverse: GGGTGGACATATAAGCGGTTC
RoRγT	Forward: CCGCTGAGAGGGCTTCAC
	Reverse: TGCAGGGAGTAGGCCACATTACA
FOXP3	Forward: CCCATCCCCAGGAGTCTTG
	Reverse: ACCATGACTAGGGGCACTGTA
β-actin	Forward: GGCTGTATTCCCCTCCATCG
	Reverse: CCAGTTGGTAACAATGCCATGT
GAPDH	Forward: CGTCCCGTAGGACAAAATGGT
	Reverse: TTGATGGCAACAATCTCCAC
HPRT	Forward: AGTCCCAGCGTCGTGATTAG
	Reverse: TTTCCAAATCCTCGGCATAATGA

### Western Blot Analysis

Cells and tissue were homogenized in lysis buffer [1 × PBS, 0.25% Trition X-100, and Pierce Protease Inhibitor Tablets (ThermoFisher)]. Forty to eighty microgram total protein was separated on 10% SDS gels. Following electrophoresis, proteins were transferred to a nitrocellulose membrane, blocked for 1 h in 5% milk with 5% goat serum, and incubated overnight at 4°C with the appropriate primary antibody, listed in Table 3. Afterwards, blots were washed 3× in 1× TBS and subsequently incubated in the appropriate fluorescent secondary antibody (1:25,000 in 5% milk; LI-COR Biosciences, Lincoln, NE) for 1 h at room temperature, washed 3× in 1× TBS and visualized on a LiCOR Odyssey. Images were analyzed in Image Studio (LiCOR).

**Table 3:** Primary antibodies used for WB, IHC, and IF staining.

**Antibody**	**Function**	**Supplier**	**Dilution**	**Application**
Anti-CD3	Detects CD3 in T-cells	Dako	1:150	IHC and IF
SMI32	Detects non-phosphorylated neurofilament H in neuronal cell bodies, dendrites, and thick axons in the CNS	Millipore	1:20,000	IHC and IF
SMI99 or SMI94	Detects Myelin Basic Protein (MBP) in the CNS	Millipore	1:10,000	IHC and IF
Anti-Iba1	Detects Ionized calcium binding adapter molecule 1 (Iba1) – a microglia/macrophage specific calcium-binding protein.	Dako	1:400	IHC and IF
Akt3 (E1Z3W)	Detects endogenous levels of total Akt3	Cell signaling	1:200	IHC
			1:1,000	WB
Anti-Mo/Rat FOXP3	Reacts with FORKHEAD BOX P3 – a transcription factor highly expressed in regulatory T-cells (Tregs)	eBioscience	1:150	IHC

### ELISA

Protein homogenates were prepared from freshly isolated corpora callosa and mechanically homogenized in 1 mL of 1 × PBS containing protease inhibitors (Pierce Protease Inhibitor Tablets, ThermoFisher Scientific). Total protein content was measured using a NanoDrop. Homogenates were subsequently spun down at 20,000 × g for 20 min at 4°C. Supernatants were collected and 100 μL were loaded into sandwich ELISA plates following manufacturer's protocol. Mouse IL-6, IL-12 p70, IFN-γ, IFN-β, IL-17, TNF-α DuoSet ELISA kits (R&D Systems, Minneapolis, MN) were used.

### Immunohistochemistry and Immunofluorescence

Immunohistochemistry (IHC) and immunofluorescence (IF) staining was done on brain and spinal cords dissected from mice perfused with 4% paraformaldehyde. Dissected tissue was kept in 4% paraformaldehyde for 24–48 h, then transferred to 25% sucrose until paraffin embedded. Paraffin-embedded sections were immersed in xylene and rehydrated through descending concentrations of ethanol and 1 × Tris buffered saline (TBS), pH = 7.4. Antigen retrieval was performed by microwaving slides in distilled boiling water, 0.01 M sodium citrate buffer, pH 6.8 (FOXP3), or 1 mM EDTA (CD3) for 7 min on high then 7 min at 70% power. Sections were then incubated in 1 × TBS containing 0.25% Triton X-100 and 3% hydrogen peroxide for 20 min at room temperature. Blocking was achieved by incubating sections in 5% nonfat milk and 5% goat serum for 1 h at room temperature. Sections were incubated with the primary antibodies in 5% non-fat milk at the specified dilutions listed in Table 3. Sections were then washed 3× with 1× TBS, 0.1% Tween20 and subsequently incubated with secondary antibody followed by incubation with the species appropriate Vecta Staining Kit (Vector Laboratories) and visualized by diaminobenzidine (DAB; Sigma). For immunofluorescent staining, fluorophore-conjugated secondary antibodies were used followed by staining with 4,6-diamidino-2-phenylindole (DAPI, 1:1,000; ThermoFisher), sections were mounted using aqueous mounting media (ProLong Gold antifade reagent; Invitrogen), and visualized using fluorescent microscopy at the indicated magnifications or scanned using Pannoramic 250 Flash III Slide Scanner (3DHistech, Budapest, Hungary).

Histological sections were graded on a scale of 0–4. For Iba1^+^ and CD3^+^ staining, cross-sectional spinal cords or brains were scored on a 0–4 inflammatory scale where a score of 0 is the equivalent pathology observed in a naïve mouse, 1 = mild inflammation, 2 = moderate, 3 = severe inflammation, and 4 = very severe inflammation involving 50% or more of the tissue (13). For relative demyelination after MBP staining, the scores were assigned as follows: 0 = MBP immunoreactivity observed in naïve mice, 1 = mild demyelination, 2 = moderate demyelination, 3 = severe demyelination, and 4 = very severe involving >50% of white matter. For relative axonal damage, we quantified the SMI32^+^ axonal swellings in the left and the right ventral region of the white matter of mouse spinal cords using multiple 20× fields. 0 = 0 SMI32^+^ axonal swellings as observed in naïve mice, 1 = ≤ 10 SMI32^+^ swellings, 2 = 10–20 SMI32^+^ swellings, 3 = 20–50 SMI32^+^, and 4 = ≥50 SMI32^+^ swellings. Slides were blinded and at least three sections of lumbar spinal cord for each animal were assessed by two individuals; the number of animals used for each experiment is indicated in the figure legends. Mann–Whitney *U*-test was used to evaluate statistical significance.

### CD4^+^ T-Cell Isolation, Activation, and Differentiation

CD4^+^ T-cells were isolated from naïve lymph nodes and spleen using anti-CD4 coupled magnetic beads (Invitrogen, Carlsbad, CA). Isolated cells were cultured in DMEM supplemented with 10% FCS, 2 mM L-glutamine, non-essential amino acids, and 50 μM β-mercaptoethanol. Cells were activated/stimulated with 0.5 μg/mL plate-bound anti-CD3e and 0.5 μg/mL anti-CD28. For T-cell activation experiments, cells were stimulated for 24 h and then collected in TRIzol for qRT-PCR analysis. For Th1 differentiation, cells were stimulated for 7 days in the presence of IL-12 (10 ng/mL; BD Biosciences), anti-IL-4 (10 μg/mL; BD Biosciences) and recombinant human IL-2 (10 U/mL; BD Biosciences). For Th17 differentiation naïve CD4^+^ T cells were stimulated for 6 days in the presence of IL-6 (50 ng/mL; Cell Signaling), IL-23 (10 ng/ml; R&D Systems), TGFβ (2.5 μg/ml; eBioscience), anti-IL-2 (10 μg/ml; eBioscience) and anti-IL-4 (10 μg/mL; BD Biosciences). Following differentiation, cells were restimulated with anti-CD3e and anti-CD28 (as above) then fixed, permeabilized and incubated with appropriate antibodies for flow cytometry; culture supernatants were collected and analyzed by sandwich ELISA using either anti-IL2-coated or anti-IFN-γ-coated plates followed by biotinylated anti-IL-2 or anti-IFN-γ secondary antibodies. Induced Treg (iTreg) were differentiated by activating naïve CD4^+^ T-cells (CD4^+^CD62L^high^) with plate bound anti-CD3 and antiCD28 antibodies for 5 days in the presence of TGFβ (2 ng/ml) and 50 U/ml recombinant human IL-2.

### Flow Cytometry

Flow cytometry was performed using a BD LSRII flow cytometer at the Albert Einstein College of Medicine Flow Cytometry Core Facility. For all experiments, single cell suspensions were prepared and resuspended in ice-cold FACS buffer [1 × PBS in 0.5% bovine serum albumin (BSA)], at a concentration of 1–5 × 10^6^ cells/mL. To prevent unspecific binding, Fc-block was performed with anti-CD16/CD32 (1 μg/mL) and incubated for 30 min on ice. Fluorochrome-labeled antibodies (0.1–10 μg/mL) to surface antigens were diluted in FACS buffer and incubated for 30 min at 4°C. Intracellular staining was performed following fixation and permeabilization where appropriate. All results were analyzed using FlowJo software (Treestar, Ashland, OR).

#### Cell Cultures

CD4^+^ T-cell cultures were surface stained with fluorochrome-conjugated antibodies anti-CD3ε (FITC clone 145-2C11) and anti-CD4 (APC-H7 clone RM4-5). For anti-IFN-γ (PE clone XMG1.2), intracellular staining was performed following permeabilization.

#### Deep Cervical Lymph Nodes (dCLN)

Single cell suspensions were prepared from the dCLN isolated from WT, CD4-Cre, CD4-CKO, and Akt3^*Nmf350*^ mice. Following Fc-block, cells were incubated with anti-CD4-Alexa700, anti-CD8-Pacific Blue, anti-CD25-PE_Cy7, anti-CD62L-APC and anti-CD44-PE. Cell subtypes were defined as follows: Tregs (CD4^+^CD25^+^CD127^−^), CD4 naïve T-cells (CD4^+^CD62L^+^CD44^−^), CD4 effector T-cells (CD4^+^CD44^+^CD62L^−^), CD8 naïve T-cells (CD8^+^CD62L^+^CD44^−^), and CD8 effector T-cells (CD8^+^ CD44^+^ CD62L^−^).

#### CNS

To evaluate the infiltrating inflammatory T cell sub-types in the CNS, the brain and spinal cord from WT, CD4-Cre, CD4-CKO, and Akt3^*Nmf350*^ mice were combined and dissociated into single cells using the Neural Tissue Dissociation Kit (T) (Miltenyl, Auburn, CA), following the manufacturer's instructions with adaptations. A 25% percoll density gradient medium was used to remove any contaminating myelin. Single cell mixtures were surface stained with anti-CD3-PE/Cy7 and anti-CD4-pacific blue. FOXP3/Transcription Factor Staining Buffer Set (eBioscience, San Diego, CA) was used for cell fixation and permeabilization prior to anti-FOXP3-APC, and-IL17-FITC, and IFN-γ-PE intracellular staining.

### Statistical Analysis

Statistical analyses were performed using GraphPad Prism software (Prism Software, Lake Forest, CA). For parametric analysis, student's *t-*test was used, and for non-parametric analysis, Mann-Whitney *U-*test and 2-way ANOVA were used. Statistical significance is presented as *p* ≤ 0.05.

## Results

### Enhanced Akt3 Kinase Activity Reduces EAE Severity

**Figure 1:**
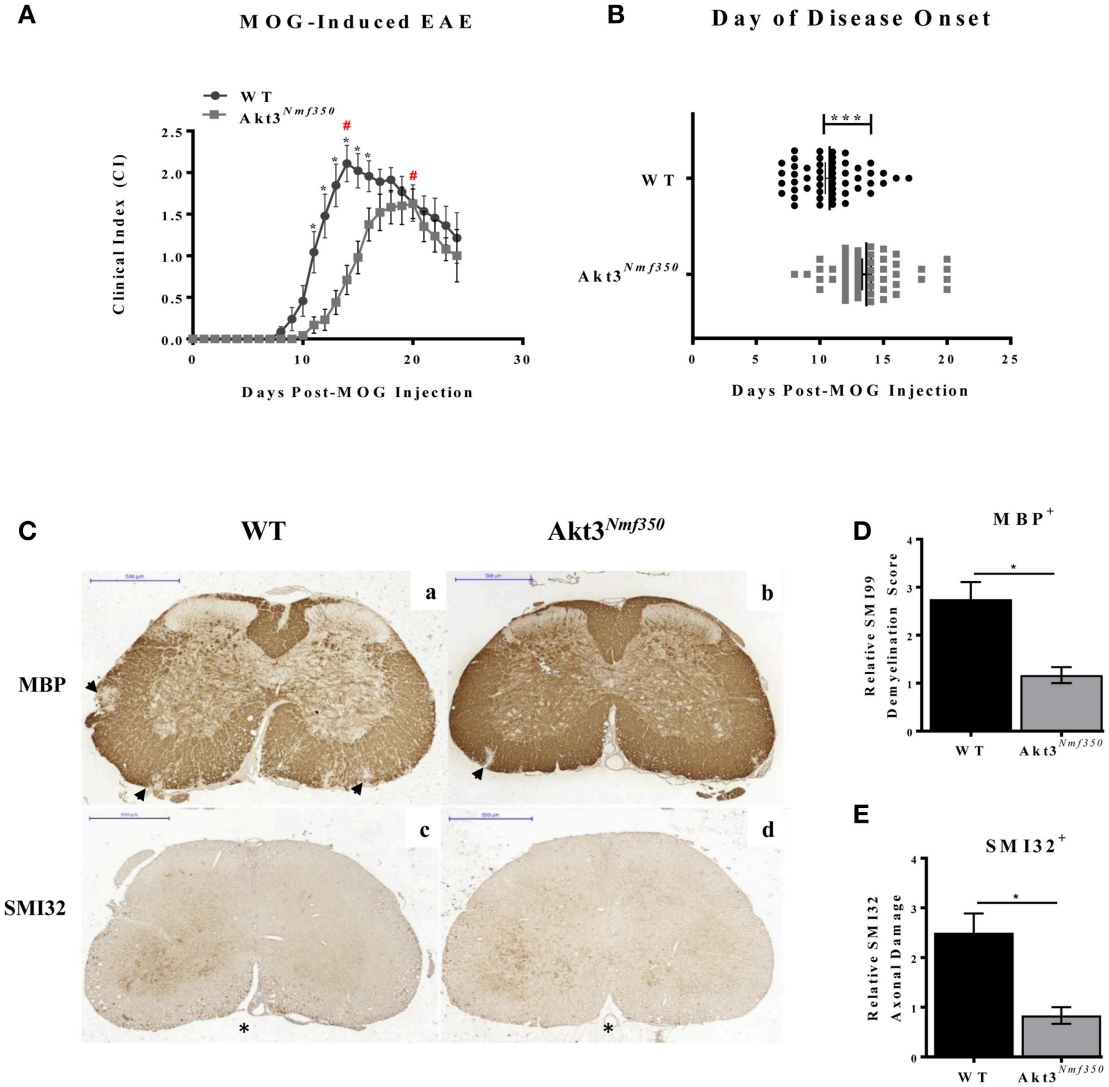
Akt3^*Nmf350*^ mice have a lag in disease onset and a less severe EAE course. **(A)** EAE disease course of male and female Akt3^*Nmf350*^ (*n* = 24) vs. WT (*n* = 24) mice. (#) Represents day of peak disease severity WT: day 14 (CI = 2.1) vs. Akt3^*Nmf350*^: day 20 (CI = 1.6) post-MOG immunization. **(B)** Day of onset of EAE symptoms (CI ≥ 1) in Akt3^*Nmf350*^ (*n* = 56) and WT (*n* = 52). **(C)** Histological analysis of representative sections of lumbar spinal cords from WT (*n* = 4) and Akt3^*Nmf350*^ (*n* = 6) mice after 10 days with consecutive clinical scores (chronic EAE) incubated with MBP (top row) and SMI32 (bottom row) are depicted. (a,b) MBP immunostaining quantified in **(D)** and (c,d) SMI32 immunostaining quantified in **(E)**. Quantification of the SMI32^+^ axonal swellings (>3 μm) was conducted on multiple 20× fields of the left and the right ventral region of the lumbar spinal cord (*p = 0.05, ***p = 0.001, Mann-Whitney *U*-test). All scale bars are 500 μm.

We have determined that a lack of total Akt3 results in a significantly worse EAE disease course (13). Therefore, to confirm the role of Akt3 during EAE we determined whether enhanced Akt3 kinase activity would reduce the severity of MOG-induced EAE. We used a mouse strain with a missense mutation, D219V, in the Akt3 gene (Akt3^*Nmf350*^) that results in enhanced Akt3 kinase activity, but does not affect total Akt3 protein levels in the brain (8). Relative to WT mice, Akt3^*Nmf350*^ mice had a less severe disease course following MOG-induced EAE including a significant lag in the onset of disease symptoms (Akt3^*Nmf350*^, 13.66 ± 0.36 vs. WT, 10.77 ± 0.34 days post-MOG, *p* < 0.0001) and significantly lower clinical scores during the acute phase of the disease (days 12 to 17 post-MOG injection; Figures 1A,B). In WT mice, the disease reached peak severity at 14 days post-MOG injection with a mean clinical index of 2.1 (CI = 2.1), whereas peak disease severity in Akt3^*Nmf350*^ mice was significantly later (20 days post-MOG), and a significantly lower clinical index (CI = 1.6), was observed (Figure 1A, #). Additionally, only 4/24 Akt3^*Nmf350*^ mice obtained a CI ≥ 2.5, compared to 11/24 WT mice. Similar results were obtained for both male and female mice, and therefore the data was pooled (Figure 1A).

Given these observations, we set out to determine whether enhanced Akt3 signaling significantly reduced demyelination and axonal damage. Paraffin-embedded spinal cord sections from WT and Akt3^*Nmf350*^ were examined at different stages of the disease, disease onset (1 day with clinical symptoms), acute EAE (4–5 days with clinical symptoms, CI ≥ 1), and chronic EAE (≥10 days with clinical symptoms, CI ≥ 1).

We first analyzed the lumbar region of the spinal cord by MBP and SMI32 immunostaining during chronic EAE. We observed significantly more demyelinated lesions that penetrated deeper into the white matter of the spinal cord of WT mice compared to that of Akt3^*Nmf350*^ mice (Figures 1Ca,b,D). Using SMI32 as a marker of axonal damage, we observed significantly more swollen axons in WT mice compared to Akt3^*Nmf350*^ (Figures 1Cc,d,E).

**Figure 2:**
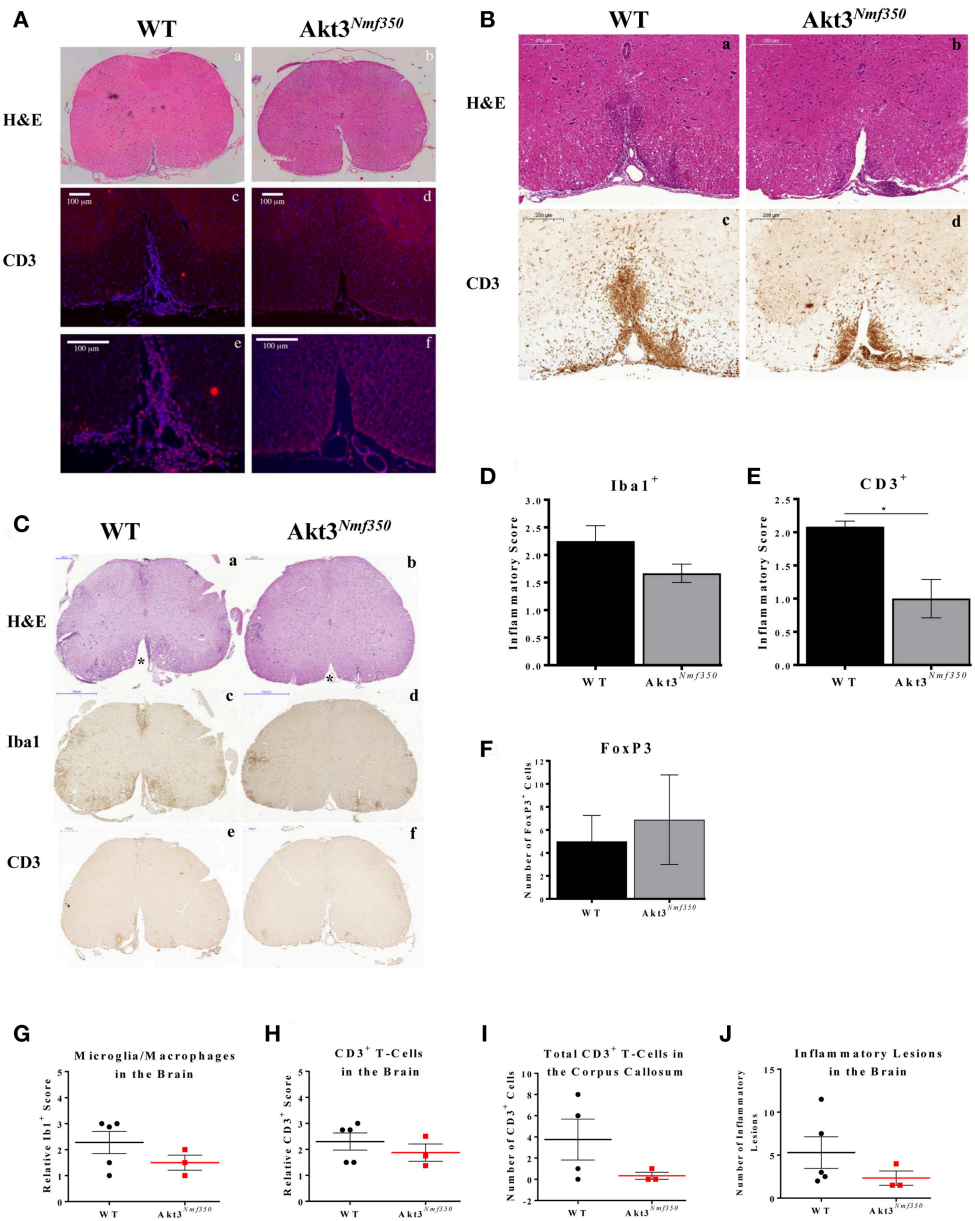
Akt3^*Nmf350*^ mice have a delay in the influx of inflammatory cells into the CNS during EAE and less CD3^+^ T-cells in the spinal cord during chronic EAE. **(A)** Representative lumbar spinal cord sections from WT (*n* = 3) (CI = 1) and Akt3^*Nmf350*^ (*n* = 5) (CI = 0) mice at 10 days post-MOG sensitization. (a,b) H&E and (c,f) CD3^+^ T-cell infiltrates (scale bar = 100 μm). **(B)** Representative lumbar spinal cord sections from WT (*n* = 3) (CI = 1) and Akt3^*Nmf350*^ (*n* = 3) (CI = 1) mice at EAE onset. (a,b) H&E and (c–f) CD3^+^ T-cell infiltrates (scale bar = 200 μm). **(C)** Representative sections from WT (*n* = 4) and Akt3^*Nmf350*^ (*n* = 6) mice after 10 days with consecutive clinical scores (chronic EAE). (a,b) H&E staining of lumbar spinal cords (scale bar = 200 μm). Asterisks (*) point to ventral funiculus. (c,d) Iba1 immunostaining quantified in **(D)** (scale bar = 500 μm). (e–f) CD3^+^ T-cell infiltrates quantified in **(E)** (scale bar = 200 μm). **(F)** Number of FOXP3^+^ cells in the lumbar spinal cord during chronic EAE, WT (*n* = 4) and Akt3^*Nmf350*^ (*n* = 3). **(G)** Quantified Iba1^+^ microglia/macrophages and **(H)** CD3^+^ cells in the brain sections of Akt3^*Nmf350*^ (*n* = 3) vs. WT (*n* = 5) mice. **(I)** Total CD3^+^ cells the corpus callosum. **(J)** Total number of inflammatory lesions in the brain (*p = 0.05, Mann-Whitney *U-*test).

To determine the contribution of the immune response to the decrease in demyelination and axonal swellings observed in the spinal cords of Akt3^*Nmf350*^ mice, we examined whether there was a concomitant decrease in the influx of inflammatory cells into the CNS. During EAE, monocytes and T-cells are activated and migrate into the CNS. Once in the CNS, activated T-cells, macrophages, and resident microglia release cytokines and inflammatory mediators that damage oligodendrocytes and contribute to neuroinflammation and myelin damage. Therefore, to examine the distribution of T-cells, we analyzed the spinal cords of both groups of mice at the onset of EAE symptoms. As there is a delay in the onset of EAE in Akt3^*Nmf350*^ mice, we first compared the spinal cords of WT mice on the first day of clinical symptoms of EAE (day 1 with CI ≥ 1), to corresponding Akt3^*Nmf350*^ mice that had not yet scored (CI = 0), ~10 days post-MOG injection. In confirmation, we also compared the spinal cords of WT and Akt3^*Nmf350*^ mice on the first day each group received a CI ≥ 1 (~10 days post-MOG injection for WT mice and ~14 days post-MOG injection for Akt3^*Nmf350*^ mice). CD3^+^ cells were detected in the ventricles of WT spinal cords (CI = 1), however no detectable CD3^+^ T-cells were detected in the ventricles or spinal cords of Akt3^*Nmf350*^ mice 10 days post-MOG injection (CI = 0) (Figures 2Ac–f). Similarly, spinal cords isolated from WT mice at the onset of EAE (CI = 1) consisted of significantly more CD3^+^ T-cells that infiltrated deeper into ventral funiculus (Figures 2Ba,b), compared to Akt3^*Nmf350*^ mice at the onset of EAE (CI = 1) (Figures 2Bc,d).

To further characterize the CNS cellular milieu, we also examined the spinal cords of mice during chronic EAE. Distinct differences were observed between WT and Akt3^*Nmf350*^ mice by H&E (Figures 2Ca,b). A significant number of infiltrating cells can be seen in the ventral funiculus of WT mice, however Akt3^*Nmf350*^ mice had no observable cellular infiltrates in that region (Figures 2Ca,b, (^*^) asterisks). In addition, although the decrease in the number of Iba1^+^ microglia/macrophages (Figures 2Cc,d,D) was not significant, we observed significantly fewer CD3^+^ cells in the lumbar spinal cord of Akt3^*Nmf350*^ mice (Figures 2Ce,f,E) compared to WT mice, consistent with Figures 2Ac–f. We measured the number of FOXP3 expressing cells in the spinal cord sections of mice during chronic EAE by IHC, and found no significant difference in the total number of FOXP3^+^ cells between the mice [WT (5 ± 2.26) and Akt3^*Nmf350*^ (6.9 ± 3.82)] (Figure 2F). However, in examining the brain sections of Akt3^*Nmf350*^ we observed a decrease in the total number of lesions, and fewer Iba1^+^ and CD3^+^ cells relative to WT mice; however, these observations were not statistically significant (Figures 2G–J).

### Akt3 Increases FOXP3 mRNA Expression and Reduces Inflammation in the Lumbar Spinal Cord During Acute EAE

**Figure 3:**
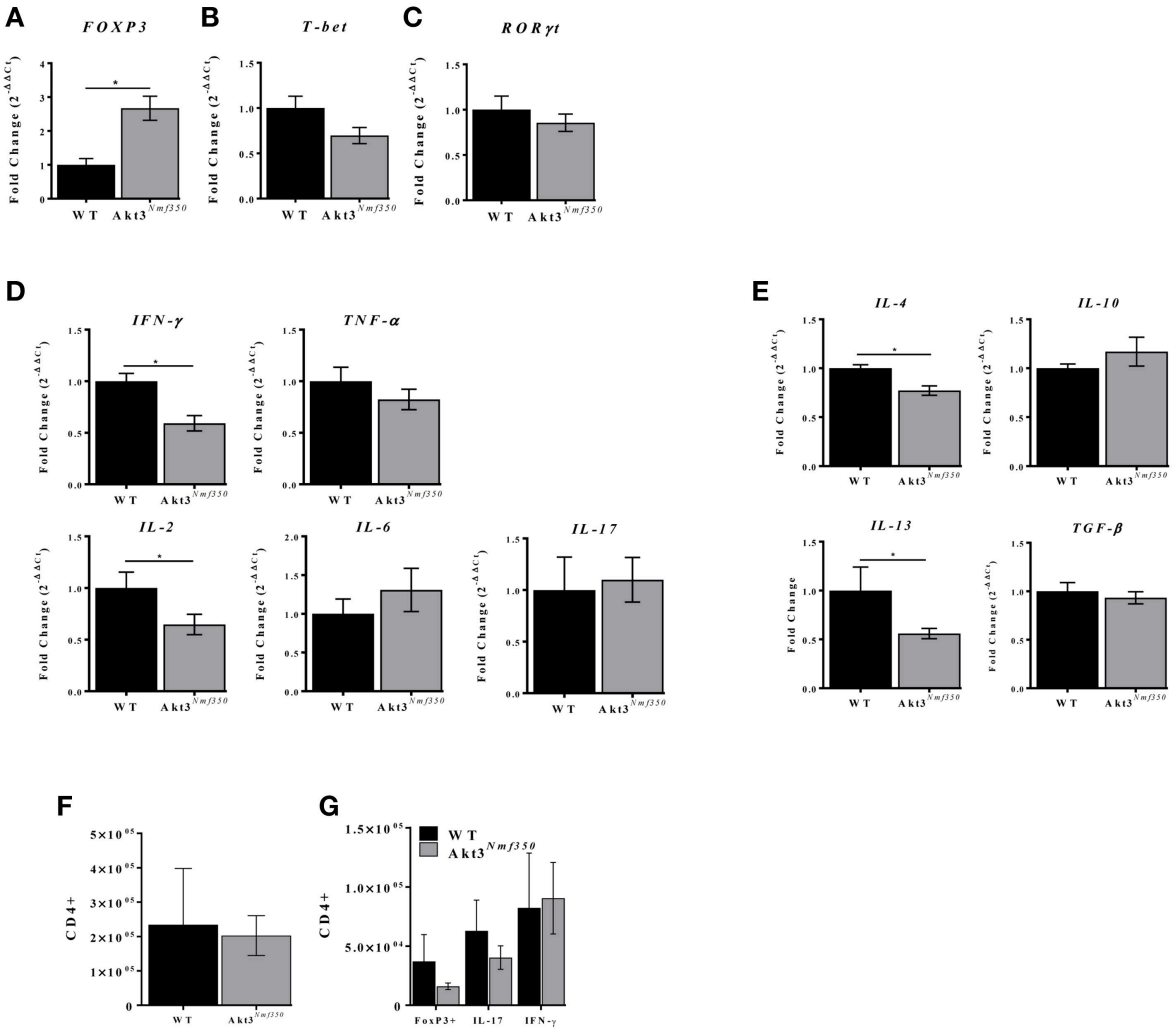
Akt3^*Nmf350*^ mice have decreased inflammatory cytokine expression and increased FOXP3 expression in the lumbar spinal cord during acute EAE. RNA was extracted from the spinal cords of WT (*n* = 4) and Akt3^*Nmf350*^ (*n* = 10) mice presenting with clinical scores for 4–5 days (acute EAE). **(A)** qRT-PCR analysis of CD4^+^ T-cell lineage-specific transcription factors: FOXP3 for Tregs, **(B)** Tbet for Th1, and **(C)** RORγT for Th17. **(D)** mRNA expression of proinflammatory cytokines TNF-α, IFN-γ, IL-2, IL-6, and IL-17; and **(E)** anti-inflammatory cytokines IL-4, IL-10, IL-13 and TGF-β. **(F)** Total CD3^+^CD4^+^ T-cells, and **(G)** CD4^+^FOXP3^+^, CD4^+^IL-17^+^, and CD4^+^IFN-γ^+^, in the CNS (brain and spinal cord combined) of WT vs. Akt3^*Nmf350*^ mice during acute EAE (*p < 0.05, Mann-Whitney *U*-test).

The mRNA expression of *FOXP3*, the master regulator of Treg development and function, was significantly reduced in the spinal cords of mice lacking total Akt3 expression (Akt3^−/−^) during acute EAE (13). Therefore, to determine the contribution of FOXP3 to the Akt3^*Nmf350*^ EAE phenotype, mRNA was isolated from the lumbar spinal cords during peak acute disease and analyzed by qRT-PCR. *FOXP3* expression was significantly elevated in Akt3^*Nmf350*^ mice compared to WT (Figure 3A). The mRNA of the Th1-related transcription factor (*Tbet*) and *ROR*γ*T* (master regulator of Th17 differentiation) were both similar to WT levels (Figures 3B,C).

To determine the extent of inflammation during EAE, we analyzed the expression of pro- and anti-inflammatory cytokines by qRT-PCR. The expression of *IL-2* and the Th1-associated cytokine *IFN-*γ was significantly lower in Akt3^*Nmf350*^ mice compared to WT (*p* < 0.05; Figure 3D). No differences were observed in the expression of *IL-6, IL-17*, or *TNF-*α. Surprisingly, Akt3^*Nmf350*^ mRNA expression of both *IL-4* and *IL-13* was significantly lower than WT, while *TGF-*β and *IL-10* remained unchanged (Figure 3E).

Since we observed an increase in *FOXP3* mRNA expression in the spinal cords of Akt3^*Nmf350*^ mice during acute EAE, we also evaluated the distribution of T-cells in the CNS (brain and spinal cord combined) during acute EAE by flow cytometry. No significant changes in the total number of infiltrating CD3^+^CD4^+^ T-cells, CD4^+^FOXP3^+^ T-cells, IL-17 producing Th17-associated T-cells, or IFN-γ producing Th1-associated T-cells were observed in the CNS (brain and spinal cord combined) of Akt3^*Nmf350*^ mice compared to WT (Figures 3F,G). Although we observed significantly fewer CD3^+^ cells in the lumbar spinal cord of Akt3^*Nmf350*^ mice compared to WT during chronic EAE (Figure 2E), the distribution of various T-cell populations in the brain and spinal cord combined were not different.

### Enhanced Akt3 Activity Significantly Increases the Number of Effector T-Cells in the Deep Cervical Lymph Nodes Before the Onset of EAE Symptoms and Increases the Number of Tregs in Draining Inguinal Lymph Nodes During Acute EAE

**Figure 4:**
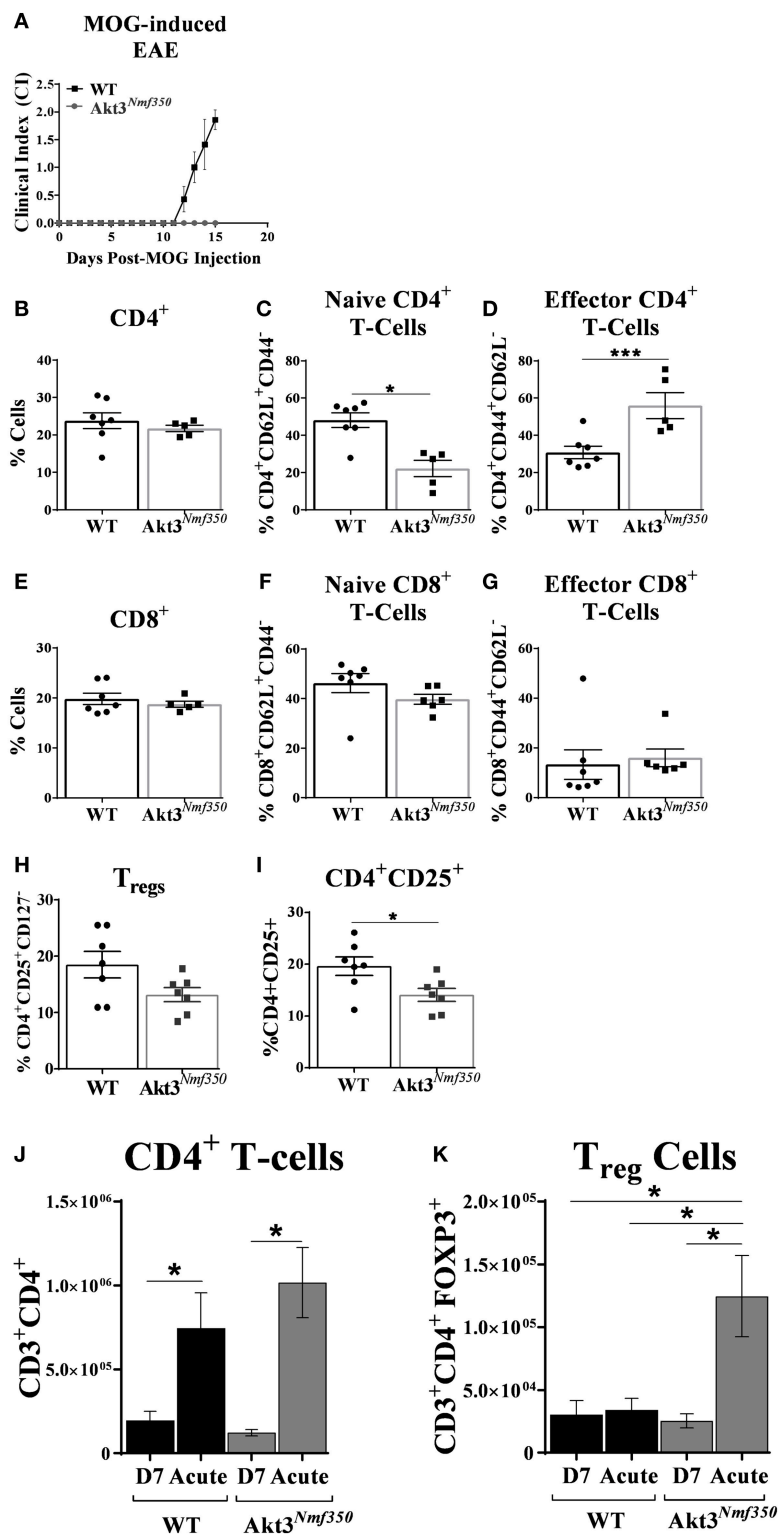
Deep cervical lymph nodes of Akt3^*Nmf350*^ mice have significantly more effector T-cells before the onset of EAE symptoms, whereas draining inguinal lymph nodes have significantly more Tregs during acute EAE. **(A)** EAE disease course of Akt3^*Nmf350*^ (*n* = 11) vs. WT (*n* = 14) mice. Mice were euthanized at 13–15 days post-MOG injection and single cell suspensions from the deep cervical lymph nodes (dCLN) were isolated for FACS analysis. **(B)** Comparison of the %CD4^+^ cells present in dCLN of WT vs. Akt3^*Nmf350*^ mice, **(C)** CD4^+^CD62L^+^ naïve and **(D)** CD4^+^CD44^+^ effector T-cell subsets. **(E)** No differences were observed in the %CD8^+^ cells, **(F)** CD8^+^CD62L^+^ naïve or **(G)** CD8^+^CD44^+^ effector T-cells in dCLN of WT vs. Akt3^*Nmf350*^. **(H)** Tregs (CD4^+^CD25^+^CD127^−^) remained unchanged; **(I)** while the percentage of cells co-expressing CD4^+^ and CD25^+^ was significantly lower in *Akt3*^*Nmf350*^. **(J)** Total CD3^+^CD4^+^ T-cells, and **(K)** CD3^+^CD4^+^FOXP3^+^ cells in the inguinal LN (iLN) of WT and Akt3^*Nmf350*^ mice during preclinical (D7 post-MOG immunization) and acute EAE (*p < 0.05, ***p < 0.001, Mann-Whitney *U-*test).

In order to accurately assess the influx of T-cells into the CNS, we harvested peripheral draining deep cervical lymph nodes (dCLN) from Akt3^*Nmf350*^ and WT mice, 15 days after MOG-sensitization (Figure 4A). Given that Akt3^*Nmf350*^ had a CI = 0 at that time point, we considered the role of Akt3 in mediating the migration of activated myelin-specific T-cells into the CNS. We observed no significant differences in the total CD3^+^, CD4^+^, or CD8^+^ T-cells between both groups of mice (Supplementary Figure 1 and Figures 4B,E). Surprisingly, there was a significant decrease in the naïve (CD4^+^CD44^lo^CD62L^hi^) T-cell population in the dCLN of Akt3^*Nmf350*^ (~22%) compared to WT (~48%) (Figure 4C). Conversely, the percentage of effector (CD4^+^CD44^hi^CD62L^lo^) T-cells was significantly higher in Akt3^*Nmf350*^ (~55%) compared to WT (~30%) dCLN (Figure 4D). No differences were observed in the CD8^+^ subtypes or Tregs (Figures 4E–G). Although we did not observe a significant decrease in the number of CD4^+^CD25^+^CD127^−^ Tregs (Figure 4H), the population of CD4^+^CD25^+^ lineage regulatory T-cells was significantly lower in the dCLN of Akt3^*Nmf350*^ mice compared to WT before the onset of EAE symptoms (Figure 4I).

Most of the lymphatics inferior to the umbilicus drain to the superficial inguinal lymph node (25); therefore, in addition to the cervical draining lymph node, we also evaluated the distribution of T-cells in the distantly draining inguinal lymph nodes (iLN) of mice by flow cytometry during the pre-clinical EAE phase (D7 post-MOG immunization) and during acute EAE. Although the total number of draining CD3^+^CD4^+^ T-cells significantly increased in both groups of mice during acute EAE relative to D7 post-MOG immunization; the number of CD3^+^CD4^+^ T-cells did not differ between Akt3^*Nmf350*^ and WT during both the pre-clinical or acute EAE phases (Figure 4J).

During the pre-clinical EAE phase, we observed no differences in the number of CD4^+^FOXP3^+^Tregs in the iLNs of Akt3^*Nmf350*^ mice compared to WT; however, during acute EAE the number of CD4^+^FOXP3^+^ Tregs significantly increased in the iLNs of Akt3^*Nmf*30^ mice relative to WT (Figure 4K). Additionally, there was an increase in the number of CD4^+^FOXP3^+^ Tregs in the iLNs of Akt3^*Nmf350*^ mice during acute EAE relative to D7 post-MOG immunization, whereas no differences were observed between the two phases in WT mice (Figure 4K). We also determined the distribution of CD4^+^CD44^+^ effector T-cells D7 post-MOG immunization, and the number of IL-17 and IFN-γ producing T-cells during acute EAE, and found no differences between the groups (Supplementary Figure 2).

### Akt3 Signaling Does Not Affect T-Cell Activation or Th1 Differentiation, but Regulates Th17 and iTreg Differentiation *in vitro*

**Figure 5:**
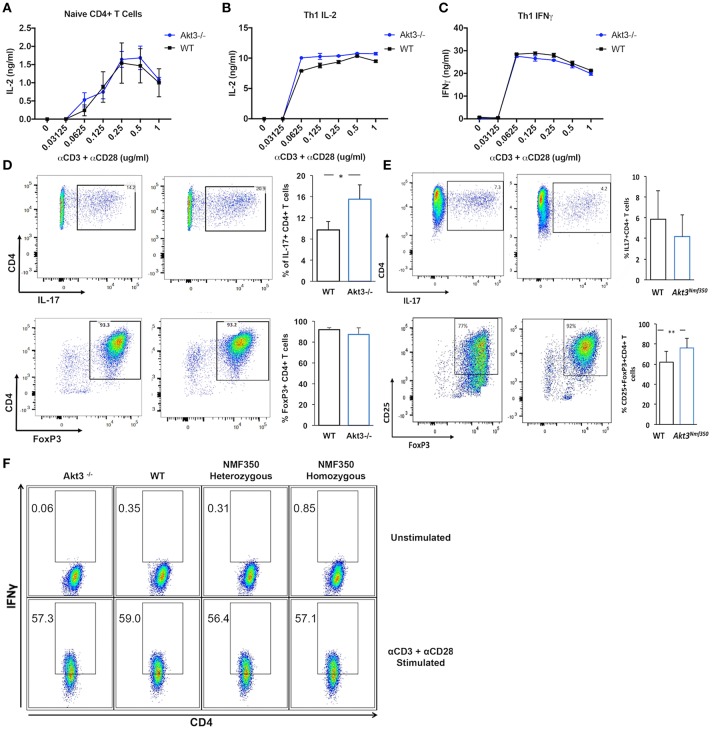
Akt3 signaling does not affect T-cell activation or Th1 differentiation, but regulates Th17 and Treg differentiation *in vitro*. **(A)** Naïve CD4^+^ T-cells isolated from cervical lymph nodes and spleens of 4–6 week old WT and Akt3^−/−^ mice were activated *in vitro* with varying concentrations of anti-CD3 and anti-CD28 antibodies for 24 h. Secreted IL-2 was measured by ELISA. **(B)** Differentiated Th1 cells from Akt3^−/−^ and WT mice were subjected to the same *in vitro* activation conditions as in **(A)**. IL-2 and **(C)** IFN-γ in culture supernatants were measured by ELISA after stimulation for 24 h. No differences in IL-2 and/or IFN-γ were detected in the culture supernatants of Akt3^−/−^ and WT T-cells. **(D)** Differentiated Th17 and iTreg cells from Akt3^−/−^ and WT mice were analyzed by flow cytometry to detect activation-induced IL-17 expression and FOXP3 levels, respectively. Representative flow cytometry data and a quantification of data collected from 3 independent experiments (mean ± SD) are presented. **(E)** Differentiated Th17 and iTreg cells from Akt3^*Nmf350*^ and WT mice were analyzed by flow cytometry to detect activation-induced IL-17 expression and FOXP3 levels, respectively. Representative flow data and a quantification of data collected from 3 independent experiments (mean ± SEM) are presented **(F)** CD4^+^ T-cells were isolated from naïve 4–6 week old Akt3^−/−^, WT, Akt3^*Nmf350*^ heterozygous, and homozygous mice using CD4 magnetic bead isolation. Cells were co-stimulated *in vitro* with anti-CD3 and anti-CD28 in the presence of IL-12 and anti-IL-4 for 7 days. Differentiated cells were restimulated in the presence of Brefeldin A for 6 h. Unstimulated and stimulated Th1 cells were then surface stained for CD4 and intracellularly stained for IFN-γ. No differences were seen in CD4^+^IFN-γ^+^ cells from any mouse strain (*p < 0.05).

Akt3^−/−^ mice have a more severe EAE disease course, and conversely, we have determined that Akt3^*Nmf350*^ mice experience a less severe disease course than WT mice. The inability to regulate T-cell activation and differentiation can lead to enhanced cytokine production and uncontrolled inflammation. To determine whether Akt3 signaling affects the T-cell activation threshold, naïve CD4^+^ T-cells and *in vivo* differentiated Th1 cells of Akt3^−/−^ and WT mice were stimulated *in vitro* with varying concentrations of anti-CD3 and anti-CD28 activating antibodies. IL-2 and IFN-γ production was measured by ELISA. Naïve T-cells from both groups of mice were fully activated with a 0.25 μg/mL concentration of α-CD3/α-CD28 (Figure 5A). No differences in IL-2 secretion were observed. Th1 differentiated cells also had similar levels of IL-2 and IFN-γ production, suggesting that that lack of Akt3 does not affect the activation of T-cells (Figures 5B,C). We further evaluated if the loss of Akt3 or enhanced Akt3 kinase activity would affect Th17 or iTreg differentiation *in vitro*. After culturing naïve CD4^+^ T-cells from WT, Akt3^−/−^, and Akt3^*Nmf350*^ mice under iTreg or Th17 differentiating conditions, we observed that although no differences were detected in the ability of Akt3-deficient CD4^+^ T-cells to become iTregs and upregulate the expression of FOXP3, cells from Akt3^−/−^ mice show a significant increased efficiency of differentiation toward IL-17 producing Th17 cells (Figure 5D). Conversely, we found a trend indicating decreased capacity of Akt3^*Nmf350*^ CD4^+^ T-cells to become IL-17 producing Th17 cells, though it did not reach statistical significance. However, a significant increased efficiency of differentiation toward FOXP3 expressing iTreg cells was observed in cells from Akt3^*Nmf350*^ mice (Figure 5E).

Given that Th1 cells are also mediators of MOG-induced EAE, we set out to determine whether Akt3 signaling can affect T-cell differentiation using *in vitro* differentiated Th1 cells from naïve Akt3*Nmf350*, Akt3^−/−^, and WT mice. The cells were analyzed by flow cytometry, and no differences in IFN-γ production were observed among the groups, suggesting that Akt3 likely does not affect the differentiation of pro-inflammatory Th1 cells (Figure 5F).

### Conditional Deletion of Akt3 in CD4^+^ T-Cells Results in an Earlier Onset of EAE

**Figure 6:**
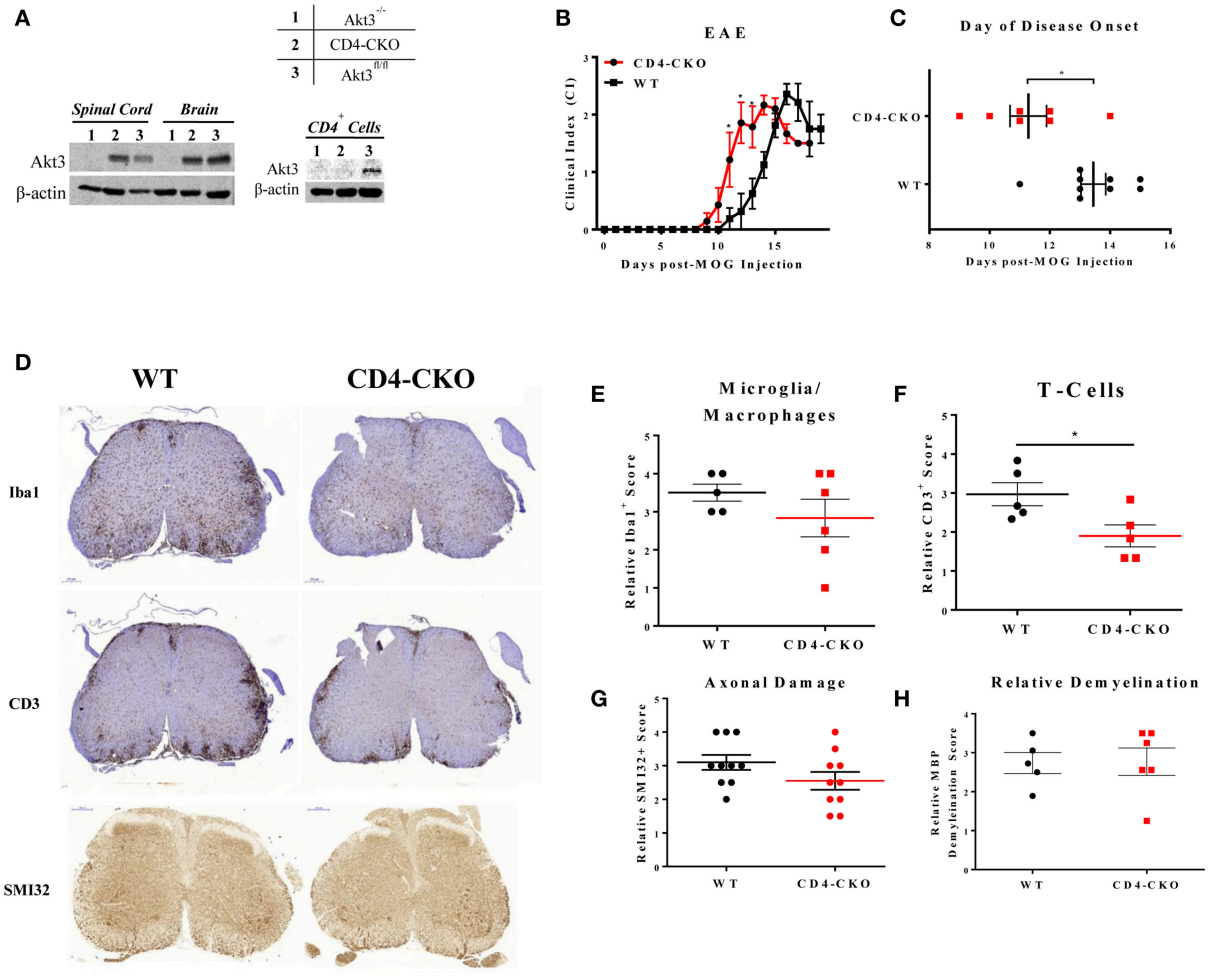
Conditional knockout of Akt3 in CD4^+^ T-cells results in earlier disease onset during MOG-induced EAE. **(A)** Western blot analysis of protein homogenates of lymph node CD4^+^ T-cells, brain, and spinal cord isolated from (1) Akt3^−/−^, (2) CD4-CKO, and (3) Akt3^fl/fl^. Densitometry analysis of CD4-CKO and Akt3^fl/fl^ brain and spinal cord homogenates yielded approximately equal ratios of Akt3/β-actin. **(B)** EAE clinical course and **(C)** day of disease onset (CI ≥ 1) of CD4-CKO mice (*n* = 7) and Akt3^fl/fl^ (*n* = 9), representative graph of 4 independent experiments. **(D)** Histological analysis of microglia/macrophages (Iba1)—quantified in **(E)**, T-cell infiltrates (CD3)—quantified in **(F)**, axonal damage (SMI32) quantified in **(G)**, and **(H)** demyelination (MBP) in lumbar spinal cord of CD4-CKO and Akt3^fl/fl^ controls after 5 consecutive days with clinical scores (*p < 0.05, Mann-Whitney *U*-test). Scale bars = 200 μm.

To specifically examine the role of Akt3 signaling in CD4^+^ T-cells, conditional knockout mice were generated using CRISPR/Cas9 technology. To generate mice with conditional deletion of Akt3 in CD4^+^ T-cells, Akt3^fl/fl^ mice were mated with CD4-Cre mice to generate CD4-Cre^+^Akt3^fl/fl^ (CD4-CKO) mice. Deletion of Akt3 in T-cells was confirmed by western blot analysis of CD4^+^ T-cells isolated from the lymph nodes and spleen of CD4-CKO mice. No detectable levels of Akt3 protein were found in the T-cells of CD4-CKO (Figure 6A, right), while Akt3 can be detected in the brain and spinal cords of CD4-CKO at similar levels to WT (Figure 6A, left). CD4-CKO and WT control mice (Akt3^fl/fl^ and CD4Cre) were sensitized with MOG and monitored. CD4-CKO mice showed signs of disease significantly earlier than WT mice with significantly higher clinical scores during the early disease phase (Figures 6B,C).

### Lack of Akt3 Expression in T-Cells Increases Inflammation in the Brains, and Decreases Tregs in the CNS of Mice During Acute EAE

**Figure 7:**
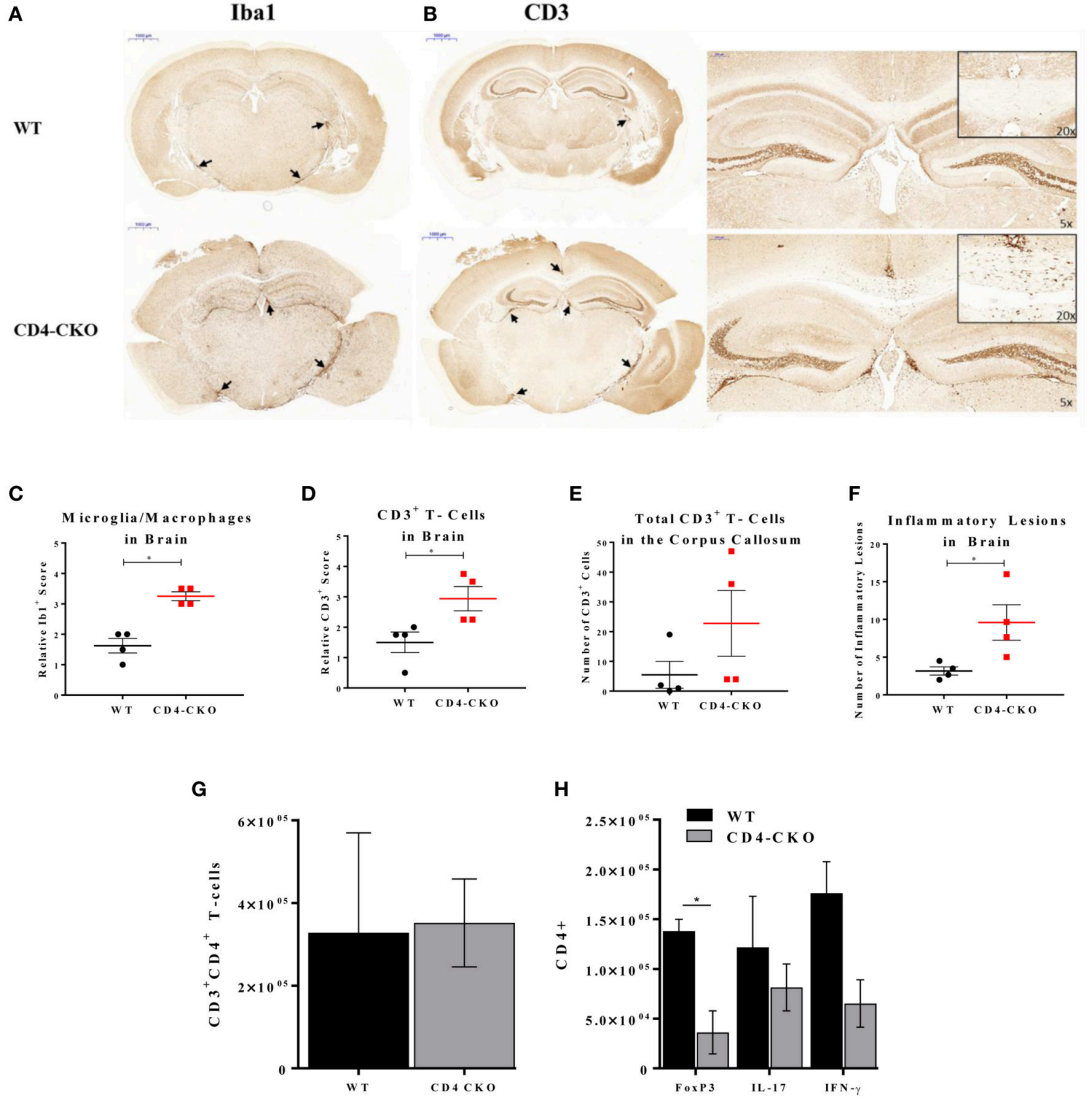
CD4-CKO mice have significantly more inflammatory lesions, CD3^+^ T-cells, and Iba1^+^ microglia/macrophages in the brain and less FOXP3^+^ Tregs in the CNS during acute EAE. Coronal sections of paraffin-embedded brains were prepared from mice sacrificed after presenting with clinical signs of EAE for 5 days (acute EAE). Immunohistochemical detection of **(A)** Iba1 (microglia/macrophages) and **(B)** CD3 in CD4-CKO and WT (Akt3^fl/fl^) mice (arrows depict lesions) (Scale bars = 1,000 μm). **(C)** Quantified Iba1^+^ microglia/macrophages, and **(D)** CD3^+^ cells in brain sections of CD4-CKO vs. WT (Akt3^fl/fl^) mice. **(E)** Total CD3^+^ cells the corpus callosum. **(F)** Total number of inflammatory lesions in the brain. **(G)** Total CD3^+^CD4^+^ T-cells, and **(H)** CD4^+^FOXP3^+^, CD4^+^IL-17^+^, and CD4^+^IFN-γ^+^ cells in the CNS (brain and spinal cord combined) of WT (CD4Cre) vs. CD4-CKO mice during acute EAE (*p < 0.05, Mann-Whitney *U-*test and one-way ANOVA).

In order to determine the function of Akt3 in T-cells during EAE, we also analyzed the brains of mice lacking Akt3 expression in T-cells during acute and chronic EAE by IHC. Although we did not observe significant differences in axonal damage, demyelination, or microglia number in the spinal cords of CD4-CKO mice compared to controls during acute disease (Figures 6D,E,G,H), the number CD3^+^ cells in CD4-CKO mice were significantly lower than that of control mice (Figure 6F). Conversely, we observed a significant increase in Iba1^+^ microglia/macrophages and CD3^+^ T-cells in the brains of the same mice during acute EAE (Figures 7A–D). Although CD3^+^ cells were clearly visible within the corpus callosum of all CD4-CKO mice, the difference in the number of CD3^+^ T-cells in corpus callosum compared to control was not significant (Figure 7B: left panel and Figure 7E). In addition, when comparing H&E, CD3, and Iba1 stained sections, CD4-CKO mice had significantly more inflammatory lesions in the brain compared to control mice (Figures 7A,B: arrows and Figure 7F).

We analyzed the distribution of T-cells in the CNS (brain and spinal cord combined) during acute EAE by flow cytometry. While no significant differences in the total number of CD3^+^CD4^+^ T-cells was observed (Figure 7G), the total number of CD4^+^FOXP3^+^ cells was significantly decreased in CD4-CKO mice relative to WT (Figure 7H). Differences in the number of IL-17 and IFN-γ producing T-cells were not significant (Figure 7H).

Additionally, peripheral T-cell repertoires were measured in dCLN and iLNs of WT and CD4-CKO mice during preclinical and acute EAE. While no differences in total CD4^+^ T cells, naïve CD4^+^ T-cells, memory CD4^+^ T-cells, and Tregs were detected in dCLNs during acute EAE, differences were observed in iLNs (Supplementary Figure 3). CD4-CKO mice displayed increased levels of total CD4^+^ T cells and FOXP3^+^ T-cells in iLNs during acute EAE relative to WT mice, and decreased levels of IL-17 and IFN-γ producing T cells (Supplementary Figure 4); however, these results did not reach statistical significance. No differences in T-cell repertoires were observed between these two groups of mice during the preclinical phase.

**Figure 8:**
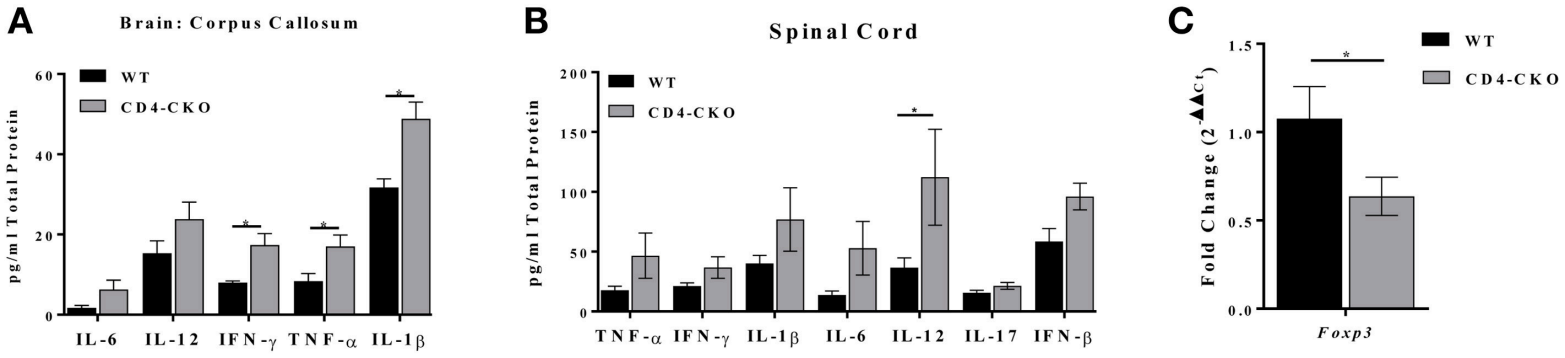
CD4-CKO mice have increased pro-inflammatory cytokine expression and decreased FOXP3 expression during chronic EAE. Protein was extracted from a 2 mm section of the corpora callosa of chronically ill mice. **(A)** Protein homogenates were prepared from CD4-CKO and WT (CD4Cre) mice, and protein levels of inflammatory cytokines measured by ELISA (pg/mg total protein) (*n* = 8–11). **(B)** Protein levels of inflammatory cytokines in the spinal cord measured by ELISA (pg/mg total protein) (*n* = 6). **(C)** Spinal cord mRNA expression of *FOXP3* measured by qRT-PCR (*n* = 6–10). All genes were normalized to HPRT expression. Differences in expression are shown as 2^−ΔΔCt^. Data were analyzed using 2-way ANOVA, *p < 0.05 is considered statistically significant.

In a parallel experiment, we measured the protein levels of inflammatory cytokines in the brains (corpus callosum) and spinal cords of CD4-CKO mice during chronic EAE by ELISA. We observed a significant increase in the protein levels of proinflammatory cytokines IFN-γ, TNF-α, and IL-1β in the corpus callosum of CD4-CKO mice relative to controls (CD4Cre) (Figure 8A). While there were no differences in the protein levels of IFN-γ, TNF-α, and IL-1β, IL-12 was significantly elevated in the spinal cords of CD4-CKO mice relative to control (Figure 8B). Although, *FOXP3* mRNA levels were undetected by qRT-PCR in the corpus callosum of both groups of mice, *FOXP3* mRNA expression was significantly reduced in the spinal cords of CD4-CKO mice compared to WT (Figure 8C).

### Conditional Deletion of Akt3 in Neurons Does Not Alter the Clinical Outcome of MOG-Induced EAE

In the brain, each Akt isoform has a unique expression pattern. Akt1 and Akt3 are primarily expressed in neurons with some expression in the hippocampus, whereas Akt2 is observed in astrocytes (26). As part of our analysis of the role of Akt3 during MOG-induced EAE, we generated a conditional deletion of Akt3 in neurons (Syn1-CKO) and examined their post-natal brain development and clinical outcome during acute and chronic EAE.

**Figure 9:**
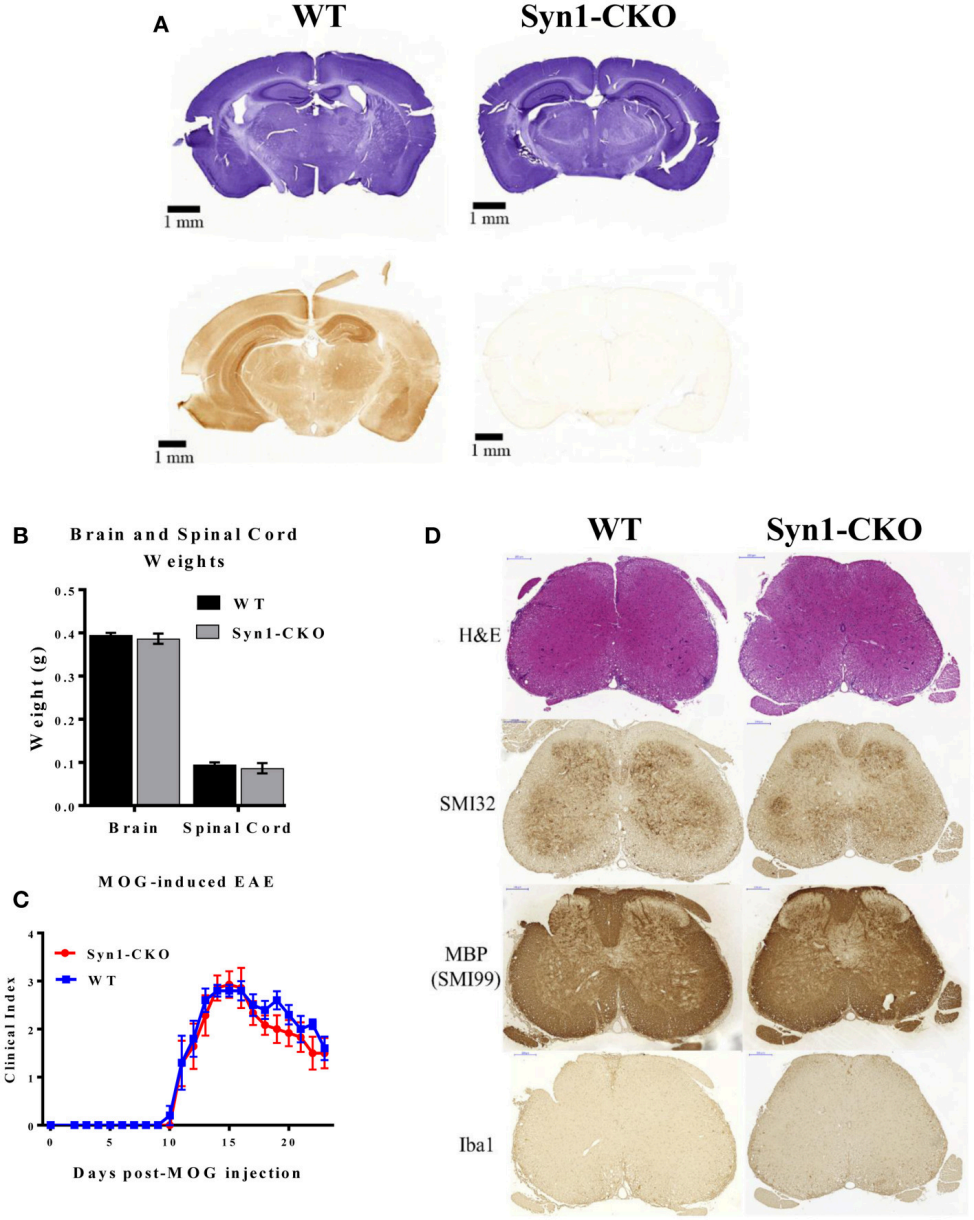
Syn1-CKO mice have similar clinical outcomes to WT mice during MOG-induced EAE. **(A)** Confirmation of Akt3 knockdown in neurons. Free-floating cross-sections of brains isolated from Syn1-CKO and WT (Syn1Cre) mice immunostained with hematoxylin (top row) or Akt3 antibody (bottom row) and visualized by DAB. **(B)** Brain and spinal cords were isolated and weighed from naïve 8–10 week old sex-matched mice (*n* = 3/group) following perfusion with 1× PBS. **(C)** Syn1-CKO (*n* = 7) and WT (Akt3^fl/fl^) (*n* = 5) mice were subjected to MOG-induced EAE. Mice were monitored daily for signs of clinical progression. No differences were observed between Syn1-CKO and WT over the course of a 23-day period following EAE induction. **(D)** From top to bottom, lumbar spinal cord sections from WT (Akt3^fl/fl^) and Syn1-CKO were stained with H&E (top), SMI32, SMI99 (MBP), or Iba1 to assess axonal damage, demyelination, and microglia/macrophage levels, respectively.

Prior to inducing EAE, naïve Syn1-CKO were examined from 6 to 10 weeks of age for brain and spinal cord weight and general cell morphology. The cellular architecture and morphology of the brain and spinal cord were similar to the naïve WT mice. Figure 9A illustrates staining of free-floating sections of WT and Syn1-CKO brains stained with hematoxylin and an Akt3 antibody. No Akt3 staining was observed in the Syn1-CKO brain. Analysis of brain and spinal cord weights was performed in naïve adult (8–10 week old) female mice following perfusion with 1× PBS. Similar brain weights were observed between Syn1-CKO and Akt3^fl/fl^ mice (0.387 ± 0.012 vs. 0.395 ± 0.005 g) (Figure 9B). In addition, there was no difference in the spinal cord weights of the Syn1-CKO and control mice (Figure 9B).

During MOG-induced EAE, Syn1-CKO mice had nearly identical clinical courses as Akt3^fl/fl^ controls, with no difference in day of disease onset (Figure 9C). Spinal cord sections of Syn1-CKO and Akt3^fl/fl^ mice during chronic EAE were analyzed by IHC. No differences in infiltrating cells (H&E), axonal swellings (SMI32), demyelination (MBP) or microglia/macrophages (Iba1) was observed between Syn1-CKO and Akt3^fl/fl^ mice (Figure 9D).

## Discussion

Although Akt3 is the most abundant Akt isoform in the brain, few studies have examined the neuroimmune effects of Akt3 signaling in the CNS. To this end, we focused our studies on determining how altered Akt3 signaling affects the clinical outcome of MOG-induced EAE. We utilized several mouse strains with different manipulations to Akt3, including mice with enhanced Akt3 kinase activity (Akt3^*Nmf350*^), mice with a conditional deletion of Akt3 in CD4^+^ T-cells (CD4-CKO), and mice with a conditional deletion of Akt3 in neurons (Syn1-CKO).

During EAE, we observed a lag in the day of disease onset and an overall decrease in severity of EAE in Akt3^*Nmf350*^ mice relative to WT mice, indicating that enhanced Akt3 kinase activity confers protection during CNS autoimmune inflammation (Figure 1). Before the onset of clinical symptoms of EAE and on the first day of physical symptoms of the disease, we observed less cellular infiltrates and fewer CD3^+^ cells present in the lumbar spinal cords of Akt3^*Nmf350*^ mice (Figure 2). As the disease progressed into the acute phase, Akt3^*Nmf350*^ mice downregulated the expression of *IL-2, IL-4, IL-13*, and *IFN-*γ relative to the levels detected in WT mice (Figure 3). By 10 days with clinical symptoms (chronic EAE), Akt3^*Nmf350*^ maintained fewer Iba1^+^ microglia/macrophages and CD3^+^ cells, with less demyelination and fewer swollen axons/spheroids, in the lumbar spinal cord; while the decrease inflammation was not significant in the brains of these mice (Figure 2).

Although IL-4 and IL-13 are generally described as anti-inflammatory, both have been implicated in promoting antibody-mediated autoimmune diseases, such as MS and EAE. Increased levels of IL-4 can be found near active demyelinating lesions of MS patients, and is elevated in the serum of MS patients during acute disease (27, 28); thus providing a rationale for the downregulation of IL-4 observed in Akt3^*Nmf350*^ mice with enhanced Akt3 signaling. IL-13 is known to promote protection during EAE (29, 30), and IL-13 producing T-cells are significantly increased in MS patients during relapse and returned to normal levels during remission (31). This production of IL-4 and IL-13 are in contrast to the genotype of Akt3^−/−^ mice during EAE, further demonstrating that Akt3 signaling confers protection during CNS autoimmune inflammation.

The development, activation, differentiation, migration, proliferation, and survival of T-cells are tightly regulated and complex processes in which Akt plays a pivotal role; and although most studies have focused on the roles of Akt1 and Akt2, data on how Akt3 may modulate T-cell function is still scarce [reviewed in (16, 32)]. Our lab has previously demonstrated that mice lacking Akt3 had an earlier disease onset and a more severe EAE clinical course. This was accompanied by increased inflammation, elevated levels of IL-2, IFN-γ, and IL-17, and fewer FOXP3^+^ Tregs in the spinal cord during EAE, suggestive of T-cell involvement. These observations led to the hypothesis that Akt3 signaling may regulate T-cell activation and/or differentiation. Therefore, we first assessed T-cell activation thresholds using varying concentrations of co-stimulatory antibodies to activate T-cells *in vitro*. We did not observe differences in the T-cell activation threshold in naïve and Th1 CD4^+^ T-cells, indicating that Akt3 signaling in these T cell populations does not affect activation.

Constitutive activation of Akt drives Th1 differentiation and upregulation of IFN-γ production in CD4^+^ T-cells in mice (33). We examined the intrinsic ability of Akt3 to regulate Th1, Th17, and iTreg differentiation *in vitro*. Upon re-stimulation following 1 week of differentiation in culture, no differences were observed in the number of CD4^+^ T-cells expressing IFN-γ. Despite not seeing any differences in Th1 or iTreg differentiation between Akt3^−/−^ and WT T-cells, there was a clear enhancement of Th17 differentiation in CD4^+^ T-cells lacking Akt3 *in vitro*. Conversely, enhanced Akt3 signaling significantly increased the efficiency of differentiation toward FOXP3 expressing iTreg cells (Figure 5). However, we cannot exclude the contribution of other populations of T-cells, as the differentiation of other T-cell populations, such as thymic Tregs, may also be affected. We also observed a significant increase in the mRNA expression of FOXP3 in the lumbar region of the spinal cord in Akt3^*Nmf350*^ mice relative to WT during acute EAE. These observations support the hypothesis that increased Akt3 signaling may lead to increase generation or recruitment of Tregs; and may at least partially account for the observed differences in clinical outcomes.

The particular T-cell functions that may be regulated by Akt3 have not been established. It is possible that inefficient suppression of effector T-cells in the periphery during the pre-clinical phase contributes to the phenotype of Akt3^−/−^ during EAE, as Akt3^−/−^ Th1 and Th17 cells are less susceptible to Treg-mediated suppression (13). However, although more efficient Treg suppression in Akt3^*Nmf350*^ mice may contribute to the delay in the development of EAE, other possibilities may include differences in T-cell migration or differentiation. The recently characterized meningeal lymphatic system and lymphatic vessels that are connected to the deep cervical lymph nodes facilitate the drainage of both fluid and immune cells from the brain parenchyma into the periphery (34). Louveau et al. (35) demonstrated that resection of the dCLN—and not the superficial CLN—delays the development of EAE and results in a milder disease pathology in mice. This suggests that dCLNs may be the site of T-cell maintenance, and that drainage of the CSF into dCLNs via the meningeal lymphatics may regulate CNS immune surveillance, a possible therapeutic target that has been largely overlooked (35). The presence of fewer naïve T-cells and elevated effector T-cells in the dCLNs of Akt3^*Nmf350*^ mice at the onset of EAE symptoms may also allude to a defect in T-cell migration from the dCLN into the CNS (Figure 4). Therefore, further characterization of Akt3 function in T-cell migration is needed to fully understand the scope of Akt3 signaling in CD4^+^ T-cells and its effects during EAE.

Given that CD4^+^ T-cells are the principal mediators of EAE pathology, and the altered T-cell function observed in Akt3^−/−^ mice, we wanted to specifically examine how Akt3 signaling in this cell population affects T-cell responses and the clinical outcome of EAE. To do this, mice with conditional deletion of Akt3 in CD4^+^ T-cells were generated and subjected to MOG-induced EAE. We observed clear differences in the day of disease onset in CD4-CKO mice relative to controls, and although we observed significant differences in the clinical index during acute EAE, these differences were not significant when normalized to the day of disease onset (Figure 6). When Akt3 signaling is enhanced in Akt3^*Nmf350*^ mice, *FOXP3* mRNA expression increased during the acute phase of the disease, although the overall number of Tregs remained unchanged (Figure 3). Conversely, we did observe fewer FOXP3^+^ Tregs in the CNS of CD4-CKO mice relative to WT mice during acute EAE (Figure 7). This was accompanied by increased cytokine expression and reduced *FOXP3* mRNA expression during chronic EAE (Figure 8). These results are consistent with our previous studies in which Akt3^−/−^ mice had fewer Tregs and increased inflammatory cytokines in the CNS during acute EAE (13).

Although the depletion of Tregs exacerbates EAE severity (36, 37) and adoptive transfer of Treg cells by systemic injection reduces EAE severity (37, 38), Tregs are known to primarily exert their immunomodulatory effects in the peripheral immune organs (39). We observed an increase in the number of FOXP3^+^ cells in the inguinal LN of Akt3^*Nmf350*^ relative to WT during acute EAE (Figure 4K). After culturing naïve CD4^+^ T-cells from WT, Akt3^−/−^, and Akt3^*Nmf350*^ mice under iTreg differentiating conditions, we determined that although no differences were detected in the ability of Akt3-deficient CD4^+^ T-cells to become iTregs and upregulate the expression of FOXP3, there was a significant increase in the efficiency of differentiation toward FOXP3 expressing iTreg cells (Figure 5D).

It was recently determined that the number of FOXP3 expressing cells in the CNS (brain and spinal cord) differ at varying time points and with disease progression (37). A study by Duffy et al. (37) showed that Treg cells (CD4^+^CD25^+^FoxP3^+^) increased in the spinal cord and brain during the clinical peak (~6 days with scores) and chronic phases of EAE (~20 days with scores) compared with the preclinical period (Day 8 post-MOG). Treg cells were also significantly higher during the chronic phase of the disease relative to clinical peak in the brain, a time point in which there is partial recovery from the disease (37). Therefore, it is probable that because Tregs intrinsically increase in the CNS of WT mice during acute EAE, any differences in Treg frequencies between WT and Akt3^*Nmf350*^ mice may only be distinct at the onset of EAE. We analyzed the distribution of T-cells in the CNS during acute EAE, and although enhanced Akt3 signaling did not increase the number of Tregs, the deletion of Akt3 in CD4 T-cells in CD4-CKO mice significantly reduced the number of Tregs in the CNS. The brain and spinal cord are distinct microenvironments that have different requirements for inflammation during EAE. IFN-γ was shown to suppress inflammation in the brain (40), yet it has a proinflammatory effect in the spinal cord during EAE (41). As a result, combining these two organs, which was necessary to obtain sufficient numbers of infiltrating inflammatory cells for flow cytometry, may have inadvertently diluted our results.

The disease course and pathology of CD4-CKO and Akt3^*Nmf350*^ during acute disease points to the notion that Akt3 signaling in CD4^+^ T-cells may largely regulate preclinical T-cell responses. One possibility is that Akt3 activity limits T-cell migration. The similar numbers of total CD4^+^ T-cells observed in the periphery and differences in the CNS, is consistent with changes in the speed in which T-cells reach the CNS following sensitization with MOG-peptide. Several cancer and developmental studies have suggested a role for Akt3 in cellular migration, and demonstrated that the downregulation of Akt3 increases migration and metastasis, and MAP-2^+^ neurons in neurospheres expressing Akt3 with a specific mutation at E17K, have a defect in migration (42, 43). Though we observed similar CD3^+^ immunostaining in the lumbar spinal cord, there was a significant increase in CD3^+^ immunostaining in the brains of CD4-CKO mice relative to controls, which suggests that Akt3 signaling in T-cells may affect migratory destinations in the CNS, which contribute to the observed EAE phenotype. Although CD4^+^ T-cells are the predominant cellular mediators of EAE, it is possible that Akt3 signaling in other immune cells like microglia, that express low levels of Akt3, may contribute to the observed disease phenotypes in our mouse models. Examination of these populations is subject for future studies.

The functional contribution of Akt3 in neurons including its role in axon growth, neuroprotection, and neuroregeneration has been noted in several studies. Constitutive activation of Akt3 in motor neurons is neuroprotective in mixed astrocyte/neuron co-cultures and in an *in vivo* model of ALS (20). The effect was shown to be mediated by the inhibition of GSK3β and ASK1, downstream substrates of Akt. In an optic nerve crush model, Akt3 was the principal Akt isoform involved in axonal regeneration via phosphorylation of GSK3β (19). These studies also found that phosphorylation of Akt3 at T305 by PDK1 was necessary for axon regeneration but phosphorylation by mTORC2 at S472 was inhibitory. Akt3^−/−^ mice have increased axonal damage during EAE, whereas Akt3^*Nmf350*^ mice have less axonal damage, leading us to hypothesize that Akt3 signaling in neurons is neuroprotective and/or neuroregenerative during CNS autoimmune inflammation.

To test this hypothesis, the synapsin 1 (Syn1) promoter was used to generate Syn1-CKO mice as it efficiently drives neuron-specific expression throughout the nervous system with widespread expression in the substantia nigra pars reticulata, motor cortex, hippocampus, cerebellum, spinal cord, ventromedial striatum, and neuromuscular junctions (44). Contrary to our hypothesis, we observed no difference in the EAE clinical course of Syn1-CKO mice, and a similar degree of axonal damage in lumbar spinal cords of Syn1-CKO mice relative to controls during chronic EAE. Whether Akt3 signaling plays a protective or regenerative role in neurons during EAE is unclear. Perhaps, long-term examination of clinical outcomes and axonal recovery may help to resolve this question. Further, Akt3 represents 30% of total Akt in spinal cord, the predominant site of neuroinflammation in the EAE model. It is feasible that Akt1 and Akt2 may have neuroprotective or neuroregenerative effects that partially compensate for the loss of neuronal Akt3 and accounts for the disease course and pathology observed in our model.

The observation that mice lacking Akt3 have an overall decrease in brain size led us to examine whether Akt3 signaling in specific CNS cell populations may contribute to this phenotype. Akt3 is highly expressed in neurons, moderately expressed in OPCs, astrocytes, and endothelial cells, and to a modest extent in microglia and oligodendrocytes (45). Our Akt3 staining of the brain (Figure 9A) suggests that neuronal expression predominates, since we observe minimal Akt3 staining in the brains of Syn1-CKO mice. Yet, other cell populations in the CNS may be affected upon global deletion of Akt3 that may play more prominent roles in post-natal brain development and the regulation of brain size. Nonetheless, we observed no differences in the weight, size, or morphology of the brain and spinal cord of Syn1-CKO mice, or differences in the clinical course of EAE compared to control mice. In confirmation of these observations, we generated another mouse strain with Akt3 deleted in neurons under the control of the CRE3 promoter (46), and observed the same EAE phenotype as that of Syn1-CKO mice (data not shown). Although Akt3 controls brain growth by regulating protein synthesis, and a reduction in ribosomal protein S6 phosphorylation is observed in Akt3^−/−^ mice (2), Levenga et al. (26) determined that only Akt1^−/−^ mice, have impaired protein synthesis dependent late-phase long-term depression (L-LTD), not loss of Akt3, suggestive of diverse functions of Akt3 in neurons.

In summary, we show that during acute EAE, increased Akt3 activity in Akt3^*Nmf350*^ mice results in a less severe EAE disease course with a lag in disease onset, delayed influx of inflammatory cells into the CNS, decreased CNS inflammation, and less axonal damage relative to controls. Conversely, CD4-CKO mice lacking CD4 expression in T-cells have an early onset of EAE symptoms with high clinical scores, less Tregs, and increased inflammation in the brain relative to control mice. However, these mice showed no differences in the pathology of the spinal cord during acute EAE disease. Additionally, Syn1-CKO mice showed no differences in EAE clinical outcomes or pathology relative to controls indicating that Akt3 signaling in neurons does not regulate axonal integrity in a CNS autoimmune setting.

These results indicate that Akt3 signaling in T-cells, and not neurons, is necessary for maintaining CNS integrity during an inflammatory demyelinating disease, and that the presence of Tregs in the CNS contributes to the dampening of EAE symptoms. Akt3 signaling plays a positive functional role in Treg differentiation; however, the exact molecular mechanisms are yet to be determined, and an extensive characterization of all the mouse models at multiple EAE phases will be required.

## Ethics Statement

This study was carried out in accordance with the recommendations of National Institutes of Health's Guide for Care and Use of Laboratory Animals. The protocol was approved by the Institute of Animal Care and Use Committee at Albert Einstein College of Medicine.

## Author Contributions

JD, AR, RG, and RA carried out experiments. JD wrote the manuscript with support from AR, RG, FM-J, and BS-Z. YZ and AR generated the new mouse models. RG, BS-Z, and FM-J provided technical support and helped interpret the results. BS-Z supervised the project. All authors discussed the results and contributed to the final manuscript.

### Conflict of Interest Statement

The authors declare that the research was conducted in the absence of any commercial or financial relationships that could be construed as a potential conflict of interest.

## Abbreviations

C57Bl6/J: strain of mice for MOG-induce EAE
CI: clinical index
CNS: central nervous system
CKO: conditional knockout
EAE: experimental autoimmune encephalomyelitis
FMCD: focal malformations in cortical development
FOXP3: forkhead box protein 3
H&E: hematoxylin and eosin stain
Iba1: ionized calcium adaptor binding protein-1
IF: immunofluorescence
IFN-γ: interferon gamma
IHC: immunohistochemistry
IL: interleukin
IP: intraperitoneal
IV: intravenous
KO: knockout
MBP: myelin basic protein
MOG: myelin oligodendrocyte glycoprotein
MS: Multiple Sclerosis
NAWM: normal appearing white matter
OPCs: oligodendrocyte progenitor cells
PI3K: Phosphoinositide 3-kinase
PNS: peripheral nervous system
Ptx: pertussis toxin
RORγT: RAR related orphan receptor C/nuclear receptor ROR gamma
RRMS: relapsing remitting Multiple Sclerosis
Tbet: T-box transcription factor expressed on T cells
TGF-β: transforming growth factor beta
TNF-α: tumor necrosis factor alpha
WT: wildtype mice, C57Bl6/J mice, Control Mice (CD4Cre, AKT3^fl/fl^ and Syn1Cre).
