# Supplementary material for: Akt3-Mediated Protection Against Inflammatory Demyelinating Disease
Source: Front Immunol. 2019 Jul 25;10:1738. doi: 10.3389/fimmu.2019.01738 (PMC6669559; doi:10.3389/fimmu.2019.01738)
Supplement: Supplementary file 1 [file Data_Sheet_1.docx]

Supplementary Material

# Supplementary Figures

**Supplementary Figure 1.** Total CD3^+^ T-cells in the CNS of **(A)** Akt3*^Nmf350^* mice and **(B)** CD4-CKO mice compared to WT during acute EAE analyzed by flow cytometry. No differences were observed. (*p < 0.05, Mann-Whitney *U-*test)

**Supplementary Figure 2. (A)** Total CD3^+^CD4^+^CD44^+^ effector T-cells in the inguinal LN (iLN) of WT and Akt3*^Nmf350^* mice during the pre-clinical EAE phase (D7 post-MOG immunization), and **(B)** CD3^+^CD4^+^IL-17^+^ and CD3^+^CD4^+^IFN-γ^+^ cells in the inguinal LN of WT and Akt3*^Nmf350^* mice during acute EAE. No differences were observed. (*p < 0.05, Mann-Whitney *U-*test)

1. **(B) (C) (D)**

**Supplementary Figure 3.** Mice were euthanized at 13-15 days post-MOG injection and single cell suspensions from the deep cervical lymph nodes (dCLN) were isolated for FACS analysis. **(A)** Comparison of the %CD4^+^ cells present in dCLN of WT vs. CD4-CKO mice, **(B)** CD4^+^CD62L^+^ naïve, **(C)** CD4^+^CD44^+^ effector T-cell subsets, and **(D)** CD4^+^CD25^+^CD127^-^ Tregs. No differences were observed in the CD8^+^ T-cell subsets. No differences were observed. (*p < 0.05, Mann-Whitney *U-*test)

**Supplemental Figure 4. (A)** Total CD3^+^CD4^+^ T-cells, and **(B)** CD3^+^CD4^+^FOXP3^+^ cells in the inguinal LN (iLN) of WT and CD4-CKO mice during preclinical (D7 post-MOG immunization) and acute EAE. **(C)** Total CD3^+^CD4^+^CD44^+^ effector T-cells in the iLN of WT and Akt3*^Nmf350^* mice during the pre-clinical EAE phase (D7 post-MOG immunization), and **(D)** total CD3^+^CD4^+^IL-17^+^ and CD3^+^CD4^+^IFN-γ^+^ cells in the iLN of WT and Akt3*^Nmf350^* mice during acute EAE. (p < 0.05, Mann-Whitney *U-*test)
